# Who’s afraid of the X? Incorporating the X and Y chromosomes into the analysis of DNA methylation array data

**DOI:** 10.1186/s13072-022-00477-0

**Published:** 2023-01-07

**Authors:** Amy M. Inkster, Martin T. Wong, Allison M. Matthews, Carolyn J. Brown, Wendy P. Robinson

**Affiliations:** 1 BC Children’s Hospital Research Institute, 950 W 28th Ave, Vancouver, BC V6H 3N1 Canada; 2 Department of Medical Genetics, University of British Columbia, 4500 Oak St, Vancouver, V6H 3N1 Canada; 3 Department of Pathology & Laboratory Medicine, University of British Columbia, 2211 Wesbrook Mall, Vancouver, V6T 1Z7 Canada

**Keywords:** X chromosome, Y chromosome, Sex differences, X-chromosome inactivation, Sex chromosomes, DNA methylation, Array, Illumina DNA methylation, Batch-correction

## Abstract

**Background:**

Many human disease phenotypes manifest differently by sex, making the development of methods for incorporating X and Y-chromosome data into analyses vital. Unfortunately, X and Y chromosome data are frequently excluded from large-scale analyses of the human genome and epigenome due to analytical complexity associated with sex chromosome dosage differences between XX and XY individuals, and the impact of X-chromosome inactivation (XCI) on the epigenome. As such, little attention has been given to considering the methods by which sex chromosome data may be included in analyses of DNA methylation (DNAme) array data.

**Results:**

With Illumina Infinium HumanMethylation450 DNAme array data from 634 placental samples, we investigated the effects of probe filtering, normalization, and batch correction on DNAme data from the X and Y chromosomes. Processing steps were evaluated in both mixed-sex and sex-stratified subsets of the analysis cohort to identify whether including both sexes impacted processing results. We found that identification of probes that have a high detection p-value, or that are non-variable, should be performed in sex-stratified data subsets to avoid over- and under-estimation of the quantity of probes eligible for removal, respectively. All normalization techniques investigated returned X and Y DNAme data that were highly correlated with the raw data from the same samples. We found no difference in batch correction results after application to mixed-sex or sex-stratified cohorts. Additionally, we identify two analytical methods suitable for XY chromosome data, the choice between which should be guided by the research question of interest, and we performed a proof-of-concept analysis studying differential DNAme on the X and Y chromosome in the context of placental acute chorioamnionitis. Finally, we provide an annotation of probe types that may be desirable to filter in X and Y chromosome analyses, including probes in repetitive elements, the X-transposed region, and cancer-testis gene promoters.

**Conclusion:**

While there may be no single “best” approach for analyzing DNAme array data from the X and Y chromosome, analysts must consider key factors during processing and analysis of sex chromosome data to accommodate the underlying biology of these chromosomes, and the technical limitations of DNA methylation arrays.

**Supplementary Information:**

The online version contains supplementary material available at 10.1186/s13072-022-00477-0.

## Introduction

﻿Many human phenotypes and diseases vary in association with sex chromosome complement and/or relative levels of gonadal hormones such as estrogens and androgens [1]. The earliest known sex differences arise when XY conceptuses grow more rapidly during preimplantation cell divisions than XX conceptuses, as reported in some in vitro fertilization studies [2–5]. Rates of certain pregnancy complications such as early-onset preeclampsia and intrauterine growth restriction also differ by fetal sex [6]. After birth, health outcomes continue to vary by sex throughout the lifespan [1]. Thus, the consideration of sex differences in genomic and epigenomic studies is essential to deepen our understanding of human health and disease, however, the study of sex as a biological variable is complicated by many factors, including the availability of validated methods for analyzing the sex chromosomes [7].

DNA methylation (DNAme) is an epigenetic modification involving the addition of a methyl group to the 5’ carbon of cytosine residues, usually in the context of cytosine-guanine dinucleotides (CpGs). In many genomic contexts, DNAme is associated with gene expression patterns, and DNAme alterations have been identified in association with numerous phenotypes and diseases. The Illumina Infinium HumanMethylation450 (450K) and MethylationEPIC (EPIC) BeadChip arrays, which assess DNAme at  > 450,000 and > 850,000 CpGs genome-wide, respectively, have been particularly popular for use in studies interrogating human DNAme. These arrays primarily assess DNAme in regions of functional relevance, such as promoters, gene bodies, CpG islands, and other regulatory regions [8]. Published DNAme analyses that investigate sex as a primary outcome variable are limited, but have demonstrated a strong signature of sex at autosomal DNAme loci [9–16]. Despite this growing body of evidence suggesting widespread sex-biased DNAme in the human genome, direct investigations into the DNAme profiles of the sex chromosomes are limited [17–22]. In fact, as many as 36% of publicly available 450K samples on the Gene Expression Omnibus public data repository do not report sample sex [23], and more often than not, sex chromosome data are excluded from processed datasets [24]; both of these factors preclude much investigation into sex differences.

DNAme array analyses of the X and Y chromosome are likely uncommon due to the analytical challenges presented by dosage inequality and X-chromosome inactivation (XCI). DNAme arrays quantify DNAme at each CpG as an average of both alleles, which has little bearing on the analysis of autosomal loci as both alleles typically have similar DNAme statuses, except at imprinted loci [25, 26]. However, in cells with more than one X chromosome, the process of XCI leads to the active and inactive X chromosomes having distinct DNAme profiles, especially at CpG island promoters where the active X (Xa) is lowly methylated and the inactive X (Xi) is highly methylated [21, 27]. Outside of gene promoters, Xi tends to have lower DNAme levels than Xa [27]. Thus, in XX samples, X chromosome DNAme quantified by array will be an average of two distinct molecular landscapes. By contrast, when the X and Y are present in single copies as in XY samples, there will be no DNAme signal averaging effect, other than theoretically in the pseudoautosomal regions if covered by the DNAme array. These differences complicate the data processing, analysis, and interpretation of X and Y chromosome results as compared to autosomal data. Analysts must be aware of how data collection by array leads to observed DNAme values, and must also consider the biological validity of downstream statistical comparisons. Similar signal-averaging effects apply to the female X chromosome signatures obtained by other genomic analysis techniques that lack allele-specificity, including but not limited to non-phased RNA sequencing, chromatin immunoprecipitation (ChIP) sequencing, and chromatin conformation studies such as Hi-C [28, 29].

Here we investigate the impact of standard processing, normalization, and batch correction steps on X and Y chromosome DNAme data. To do this we assembled a cohort of Illumina Infinium HumanMethylation450 array data from 634 normative term human placentas from public datasets with raw data available in the form of IDAT files. We determine which processing steps should be done differently or in a sex-stratified manner when working with sex chromosome data. We also develop a set of biology-informed recommendations for X and Y chromosome DNAme data analysis, for which the analytical method of choice depends on the research question under study. We tested this framework by interrogating differential X and Y chromosome DNAme in acute-chorioamnionitis-affected placental samples. Our findings apply to both 450K and EPIC array data as the underlying probe chemistry is shared, and are also generalizable beyond the tissue investigated (placenta) as XCI is a pan-tissue process.

## Results

### Sex mismatches or unexpected karyotypes should be identified and removed from datasets before processing and analysis

This study was conducted on human placental samples. As an organ originating from cells of the conceptus, the placenta shares the same genotype as the fetus and can be treated similarly to other single-donor tissues measured with Illumina’s DNAme arrays. When working with placenta, though, it is necessary to apply sampling methods designed to reduce the potential for contamination with maternal tissue, such as sampling from the fetal side of the placenta and washing thoroughly to remove any traces of maternal blood. For a description of how maternal contamination is avoided in sampling, see Methods. Contaminated samples can further be rigorously identified and removed from public datasets using the 65 SNP genotyping probes present on the 450K array. In a cohort of 711 placental samples with 450K array data, 72 samples were excluded for possible contamination using these 65 genotyping probes; see Additional file 1: Figure S1. Similar genetic contamination checks should be done in all tissues regardless of origin, but are particularly important when working with prenatal tissue.

Confirming that sex chromosome complement matches the metadata-annotated sex is a critical first step when the sex chromosomes will be analyzed downstream. Imputing sex from DNAme array data can also serve as a valuable quality control step to identify unintentional sample mix-ups during processing, inter-sample contamination, or biological conditions including unknown aneuploidy or disorders of sexual development; this step is further useful when sample sex is not annotated. Several sex-imputation tools are available for use with Illumina DNAme array data; see Additional file 1: Table S1. In the present dataset, we removed four samples from the dataset as the metadata disagreed with data-derived sex. One additional sample was identified to be likely mosaic for both 45,X and 46,XX cells and was also removed; see Additional file 1: Figure S﻿1. The demographics for the final cohort of 634 samples are presented in Table 1.

**Table 1:** Cohort demographics

	Female (XX)	Male (XY)	p-value*
*N*	309	325	
Gestational age [weeks, mean (SD)]	39.43 (1.12)	39.46 (1.05)	0.663
Cohort (*n*)			0.770
EPIC^†^	25	28	
NHBC (GSE71678)	144	159	
RICHS (GSE75248)	140	138	
PlaNET ancestry			
Coordinate 1	0.28 (0.21)	0.28 (0.18)	0.968
Coordinate 2	0.37 (0.25)	0.37 (0.21)	0.780
Coordinate 3	0.36 (0.25)	0.35 (0.22)	0.759

Demographics of the 634-sample cohort with Illumina Infinium HumanMethylation450 BeadChip data in IDAT format, used to evaluate effect of processing and analysis on X and Y chromosome DNAme signatures

^*^p-values are from Fisher’s exact test for categorical variables and t-tests for continuous variables

^†^EPIC normative term samples deposited in projects GSE100197, GSE108567, GSE98224, and GSE74738

### Average XY beta values vary with chromosome complement

**Fig. 1:**
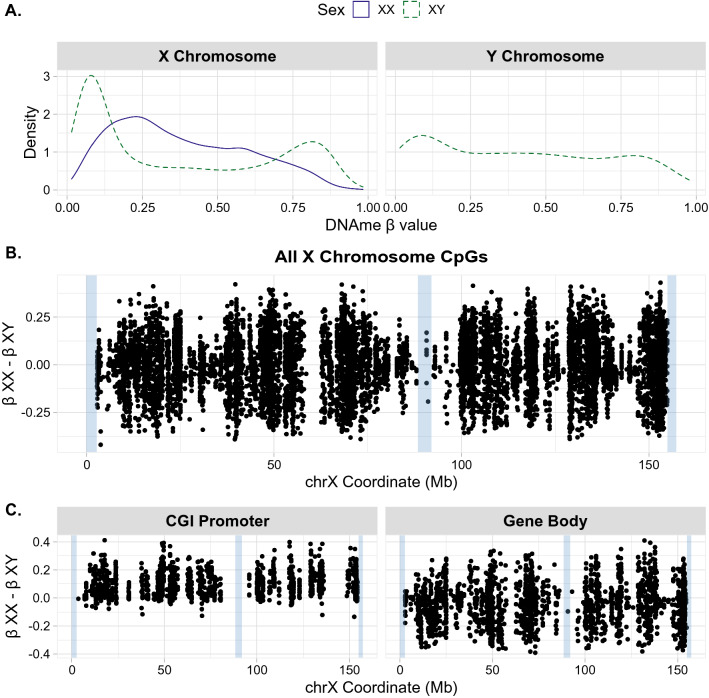
DNA methylation profiles of the X and Y chromosome. All plots show raw (non-normalized) data after probe filtering. **A** Density plot of X and Y chromosome beta values by sex. XX sample beta value densities are shown in blue, XY sample beta value densities are shown in dashed green. **B** Difference in DNAme between XX and XY samples at all CpGs along the X chromosome in the filtered dataset, difference in DNAme *β* values between the sexes (XX-XY) is plotted on the Y axis, hg19 X chromosome coordinates are plotted along the x axis. Blue highlights indicate PAR1 (chrX:60001–2699520), the X-transposed region (chrX:88400000–92000000), and PAR2 (chrX:154931044–155260560). **C** Difference in DNAme between XX and XY samples along the X chromosome using hg19 chrX coordinates. Plots are separated into CpGs associated with CpG island (CGI) promoter or gene body regions, blue highlights indicate PAR1, the X-transposed region, and PAR2

The overall distribution of DNAme on the X chromosome showed chromosome-wide sex differences, as expected. The X in XX samples exhibited higher average CpG island promoter DNAme than in XY samples, representing CpGs that are highly methylated on Xi and lowly methylated on Xa, typical of promoters undergoing XCI-mediated DNAme. At the promoters of genes silenced by XCI, the combined signature of Xa and Xi DNAme has previously been termed “MeXiP” for Methylation of Xi Promoters [21]. At gene bodies, the average DNAme across the X chromosome was more similar between XX and XY samples, see probe-filtered, non-normalized DNAme distributions in Fig. 1.

The landscape of Y chromosomal DNAme in XY samples exhibited a range of DNAme beta values; see Fig. 1. While there was a peak at low DNAme values, this was less pronounced than seen for autosomes or for the X in XY cells and there was also not a clear peak at high DNAme values. A similar pattern was observed in blood, but with a slightly larger hypermethylated peak, likely owing to the fact that placenta tends to have less DNAme than other tissues; see Additional file 1: Figure S2 [30]. In general, the Y chromosome may exhibit more DNAme inter- and intra-individual variability, which could be an interesting area of future investigation.

### Identifying probes for removal with high detection p-value, but not low bead count, should be sex-stratified for the Y chromosome

Probes on Illumina’s DNAme arrays with a high detection p-value or low bead count are typically identified and removed from datasets, as these metrics indicate poor probe performance [31, 32]. In XX samples, Y chromosome probes are expected to have higher average detection p-values than in XY samples, because in the absence of Y chromosome genetic material these probes will only measure background fluorescence (high detection p) [31, 33].

**Fig. 2:**
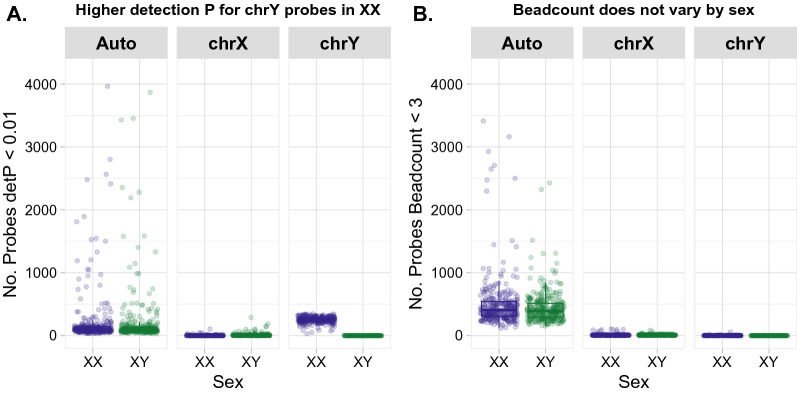
Probes failing detection p-value but not bead count steps vary by sex and chromosome. **A** Significantly more Y chromosome probes have high detection p-values on average in XX (blue) as compared to XY (green) samples. No sex difference in detection p-values was observed at autosomal or X chromosomal probes. **B** No sex difference was observed between bead counts of probes from the autosomes or X/Y chromosome

We found that probe filtering in a mixed-sex cohort led to excessive removal of Y chromosomal probes with high detection p-values (359/416, or 86%), as compared to the proportion of probes removed from the autosomes or X chromosome (1.1% and 1.3%, respectively). However, when Y chromosome detection p-values were assessed in only XY (male) samples, the proportion of Y chromosome probes failing at this stage (0.72%) was consistent with the proportion of autosomal probes that fail detection p-value filtering; see Fig. 2 and Table 2. We found no difference in X chromosome filtering of high detection p-value probes with sex-stratification as compared to a mixed-sex cohort (*n* = 4,979, and *n* = 4,979). Therefore, we recommend that probe filtering by detection p-value always be conducted in a sex-stratified manner for the Y chromosome in mixed-sex cohorts.

**Table 2:** Number of probes indexed as failing during detection p-value, bead count, and non-variable probe checks

Probe filtering step	Chromosome	*n* probes on 450k	*n* probes filtered (full cohort)	*n* probes filtered (sex-stratified)	Full cohort versus sex-stratified p-value*	Comment
Detection p-value	Auto	473.864	4979	4979	< < 0.00001	Sex-stratify
	X	11.232	143	143		
	Y	416	359	3		
Bead count	Auto	473.864	4805	4805	0.76	Do not stratify
	X	11.232	94	94		
	Y	416	3	1		
Non-variable	Auto	473.864	154.976	154.976	0.0005	Sex-stratify
	X	11.232	725	725		
	Y	416	0	79		

Detection p-value indexes probes with a detection p > 0.01 in > 1% of samples, bead count refers to probes with a bead count of  < 3 in > 1% of samples, and non-variable refers to the number of probes with a range of DNAme beta values of  < 5% across samples

^*^p-values from Fisher’s exact tests for categorical variables

Bead count is another commonly used quality control metric, and probes with a fluorescent signal reported by < 3 beads in more than a user-defined percentage of samples are commonly removed from datasets prior to analysis [32, 34]. We did not expect bead count calling to depend on the chromosome under study, as the ability of a probe to be detected by the scanner is independent of its fluorescence signal or hybridization. We found that the relative proportions of low bead count probes did not differ for the X and Y chromosome by sex, confirming that there was no need to stratify by sex during this filtering step; see Table 2 and Fig. 2.

### Non-variable probe filtering should be sex-stratified for the Y chromosome

Non-variable probes, or those with very similar DNAme profiles across all samples within a cohort, are often removed in DNAme analyses as it is assumed that they will be uninformative in downstream differential methylation testing. The removal of non-variable probes is advantageous as it reduces the multiple test correction penalty [35].

We hypothesized that non-variability calling would need to be stratified by sex for the Y chromosome as the Y chromosome beta value in XX samples does not reflect DNAme status. When indexing the variability of beta values per probe, Y chromosome probes in XX samples show average beta values of roughly 0.5, as both the methylated and unmethylated channels have similarly near-zero fluorescence at Y chromosome probes, and beta values are calculated as the ratio of methylated over total intensity. In contrast, in XY samples, the Y chromosome displays a range of beta values. Accordingly, in our mixed-sex cohort no probes on the Y chromosome were non-variable across all samples. In contrast, when assessing non-variable Y chromosome probes in XY samples alone, multiple probes qualified as non-variable on the Y chromosome (79 probes, 19% of chrY probes), see Table 2; this was similar to the proportion of non-variable probes identified on the autosomes and X chromosomes (Fisher’s exact test, p > 0.05). Thus, we concluded that the identification of non-variable probes on the Y chromosome should be assessed only in XY samples. The subsequent step of overlapping a cohort-specific list of non-variable probes with independent probe exclusion lists can then be performed.

#### Non-specific and polymorphic probe databases vary widely in coverage of X and Y chromosome

Several published lists exist to index probes on Illumina’s DNAme arrays that should be removed due to design flaws (underlying polymorphisms or potentially non-specific probe sequences) [33, 36–38]. We investigated several common non-specific probe resources, and found that (i) all indexed probes for removal from the X and Y chromosome, and (ii) the magnitude of X and Y non-specific probes indexed by each resource was quite similar; see Table 3. As such, we find that non-specific probes can be indexed on the X and Y using the same probe exclusion lists usually applied to the autosomes. In contrast, the coverage of the X and Y chromosome varies widely among commonly used polymorphic probe exclusion lists; see Table 3. This discrepancy between resources appears attributable to the version of SNP database used to create the polymorphic probe annotation, with more recent versions providing better X and Y chromosome coverage. The overlaps between the multiple resources indexing non-specific and polymorphic probes are presented in Additional file 1: Figures S3 and S4. Accordingly, we recommend using the most updated polymorphic probe exclusion lists available when working with X and Y chromosome DNAme data to ensure comprehensive sex chromosome coverage. Alternative methods to probe exclusion lists are also available; see Discussion.

**Table 3:** ﻿X and Y chromosome coverage of common resources indexing non-specific probes, and polymorphic probes

Annotation	Citation	Definition of non-specificity	X	Y
450K array non-specific probe annotations				
Zhou et al. 2017	[36]	High-quality sequence match to intended target (based on internal score), with unique 30 nucleotide 3ʹ subsequence (MASK.mapping & MASK.sub30.copy)	1267	174
Price et al. 2013	[37]	> 40 nucleotide match with > 90% identity and no gaps, must match at position 50	1202	146
Chen et al. 2013	[38]	≥ 47 nucleotide match with no gaps and a match at position 50, only best match retained	819	116
EPIC array non-specific probe annotations				
Zhou et al. 2017	[36]	High-quality sequence match to intended target (based on internal score), with unique 30 nucleotide 3ʹ subsequence (MASK.mapping & MASK.sub30.copy)	2018	217
Pidsley et al. 2016	[8]	≥ 47 nucleotide homology with at least one off-target locus	962	221

Annotation	Citation	SNP reference	Definition of polymorphic	X	Y
450K array polymorphic probe annotations					
Zhou et al. 2017	[36]	dbSNPv147 + 1KGP.phase3	MAF > 0.01 within last 5 nucleotides of probe including CpG or SBE, or SNPs that cause a color switch for Type I probes. (MASK.extbase, MASK.typeINextBaseSwitch, & MASK. snp5.GMAF1p)	145	2
Illumina	[84]	dbSNPv147	Polymorphism at CpG or SBE (MAF > 0.01)	181	0
Illumina	[84]	dbSNPv132	Polymorphism at CpG or SBE (MAF > 0.01)	48	0
Price et al. 2013	[37]	dbSNPv131	Polymorphism at CpG (any MAF, heterozygosity > 0.1 greater effect)	164	9
Chen et al. 2013	[38]	1KGP.phase1	Polymorphism at CpG (any MAF)	0	0
EPIC array polymorphic probe annotations					
Zhou et al. 2017	[36]	dbSNPv147 + 1KGP.phase3	MAF > 0.01 within last 5 nucleotides of probe including CpG or SBE, or SNPs that cause a color switch for Type I probes. (MASK.extbase, MASK.typeINextBaseSwitch, & MASK. snp5.GMAF1p)	253	7
Illumina	[84]	dbSNPv147	Polymorphism at CpG or SBE (MAF > 0.01)	166	0
Illumina	[84]	dbSNPv132	Polymorphism at CpG or SBE (MAF > 0.01)	45	0

SBE indicates single base extension site

The common polymorphic probe resources listed in Table 3 do not index many polymorphic probes on the Y chromosome, particularly those resources based on older SNP references. To test whether this is an artifact of substandard coverage of the Y chromosome by common SNP resources, we downloaded the complete list of Y chromosome SNPs (*n* = 611 at all allele frequencies) from the Y Chromosome Consortium [39] and overlapped the hg19 coordinates for these loci with the 50-nucleotide hg19 coordinates of the 450K and EPIC Y chromosome probes. We found that none of the Y Chromosome Consortium SNPs overlapped with any Y chromosome probe on either the 450K or EPIC arrays, and more importantly, none overlapped within the critical last five base pairs of a probe sequence, which Zhou et al. found to be most sensitive to technical interference by sequence polymorphisms [36]. As such, polymorphic probe annotations appear to be accurate for the Y chromosome despite few probes being indexed for removal as compared to the autosomes or X chromosome; this may change in the future as more SNPs are indexed on the Y chromosome [39].

### Other probe types on the X chromosome that can be removed based on the question of interest

When working with autosomal DNAme array data, probe filtering is typically restricted to poor quality, polymorphic, and non-specific probes. However, given the evolution of the sex chromosomes from a pair of homologous autosomes, the X and Y chromosomes have very high sequence identity at several regions, coupled with distinct sex differences in DNAme profiles. The sequence similarity means that more probe categories should be considered for removal when working with X and Y chromosome data from the 450K and EPIC arrays. We first investigated how many probes targeted regions of high homology between the X and Y, including the pseudoautosomal regions (PAR) and the X-transposed region (XTR) [40, 41]. Using PAR coordinates from the UCSC hg19 build, we found that no probes on either the 450K or EPIC array targeted PAR1 or PAR2 sequences on either the X or Y chromosome. These PAR regions seem to have been excluded entirely from the Illumina DNAme array platforms. However, 25 probes (X chromosome) and 31 probes (Y chromosome) targeted the XTR on the 450K array; see Table 4.

**Table 4:** Probe coverage of the 450K and EPIC arrays in areas of high X–Y chromosome homology

Category	Coordinates (hg19)	Coordinate source	*n* Probes 450K	*n* Probes EPIC
PAR1 chrX	60001–2699520	[85]	0	0
PAR2 chrX	154931044–155260560	[85]	0	0
PAR1 chrY	10001–2649520	[85]	0	0
PAR2 chrY	59034050–59363566	[85]	0	0
XTR chrX	88400000–92000000	[41]	25	59
XTR chrY	3440000–5750000	[41]	13	31

Most probes that overlapped the XTR on the 450K array were reported to be non-specific in Zhou et al. [36] (31/38), Price et al. [37] (27/38) and Chen et al. [38] (27/38) [36–38]. All probes on the 450K and EPIC arrays that overlap the XTR are indexed in Additional file 2: Table S2 and Additional file 3: Table S3 if users wish to evaluate them for non-specificity in particular datasets with packages such as *UMtools* [33], or to investigate their DNAme patterns post hoc. We suggest that if these probes arise in differential DNAme analyses, users should BLAT or BLAST [42] the individual probe sequences to identify and report on the confidence of the hybridization locations.

Probes in the promoters of cancer testis genes should also be considered for removal when working with X chromosome data, particularly if users want to evaluate XCI by considering promoter DNAme levels [22, 43]. Members of the cancer-testis gene family are normally expressed only in testis or cancer cells, and these genes tend to have high promoter DNAme in all other tissues in both sexes [44]. As many cancer testis genes are located on the X and Y chromosomes, Cotton et al. [43] recommended removing these probes if evaluating X-chromosome inactivation as their promoter DNAme level does not generally correlate with XCI status [43]. We overlapped the 450K and EPIC probe coordinates with a complete list of cancer testis gene locations indexed by the Cancer Testis database (http://www.cta.lncc.br/) and identified 553 (450K) and 622 (EPIC) X and Y chromosome probes that target the promoters of cancer testis genes and should be considered for removal; see Additional file 2: Table S2 and Additional file 3: Table S3.

Finally, repetitive elements are abundant in the genome, and are relevant to XCI. Long interspersed nuclear elements (LINE repeats), in particular, are roughly twofold more abundant on the X chromosome than in the autosomal genome [45]. Similar to the cancer testis probes, in studies of XCI by promoter DNAme, CpGs in repetitive elements are typically excluded from analyses due to cross-hybridization potential [43]. Considering all classes of repetitive elements indexed by RepeatMasker [46, 47], we identified 688 (450K) and 1975 (EPIC) probes, respectively, that overlapped repetitive elements on the X and Y chromosome. Many of these probes are also indexed as non-specific in common resources [36–38], though not all. We provide an annotation for all X and Y chromosome probes indicating whether they overlap repetitive elements, and what type/family of repetitive element is overlapped for both the 450K and EPIC arrays in Additional file 2: Table S2 and Additional file 3: Table S3. For probes in repetitive elements that are not flagged for removal by non-specific probe annotations, we recommend a similar process as suggested for treatment of XTR probes: if these probes arise as significantly differentially methylated in association with a phenotype of interest, users should report BLAT or BLAST results to provide transparent estimates of probe specificity.

#### Normalization effect on X and Y chromosome DNAme distributions does not differ between algorithms

Normalization algorithms attempt to harmonize the data from the Infinium I and Infinium II type probes included on the 450K and EPIC arrays [11, 48, 49]. Users must choose between several available normalization algorithms based on parameters such as the existence of known technical batch effects, or the number of tissues included in a study [50]. As normalization transforms beta value distributions, it has been posited that sex differences in the distribution of X and Y chromosome beta values may interact with normalization [51]. Here, we assessed the extent to which raw X and Y chromosome data (pre-normalization) differed from data post-normalization to assess potential damage inflicted by unsuitable normalization procedures, and to understand whether the impact on X and Y chromosome data should be an important consideration in the choice of normalization algorithm.

We applied seven commonly used normalization algorithms to our full dataset: functional (with and without noob) [52], beta-mixture quantile (BMIQ) (with and without noob) [53], dasen (with and without noob) [32], and noob alone [54]. Each of these algorithms was applied to the full dataset. For each sample, we calculated Spearman’s rho and the root mean square error (RMSE) of (i) the X chromosome and (ii) the Y chromosome beta values before versus after normalization.

For both the X and Y chromosome, all methods returned high Spearman correlation coefficients and low RMSE values between raw and normalized DNAme values; see Additional file 1: Figure S5 and Table S4. For the female X, all normalization methods yielded high intrasample correlation values between 0.9959–0.9996; for the male X the range was 0.9842–0.9994, for the Y, the range was 0.9933–0.9979. The RMSE ranges were very similar and low for all normalization methods: female X 0.023–0.059, male X 0.019–0.068, Y 0.033–0.063. These findings support overall similarity between methods and suggest that between-algorithm effects of normalization on X and Y chromosome DNAme distributions may not need to be a primary consideration when selecting a normalization method. See Discussion for a further commentary on the selection of normalization algorithm in analyses of autosomal sex differences or the X and Y chromosome.

### Batch correction with sva ComBat does not substantially differ with sex-stratification

Several methods have been proposed to correct DNAme data for systematic technical variation or batch effects, most of which rely on statistical adjustment or estimation and removal of batch effects prior to statistical analyses, using tools such as *sva* ComBat [55, 56]. Proper use of ComBat requires (i) provision of a model describing all variables of interest, and (ii) application to only balanced datasets where batch variables are not confounded with variables of interest [57–59]. In studies where the X and Y data will be analyzed, sex must be considered as a potential confounding variable in all applications of ComBat batch correction. We therefore sought to evaluate whether X and Y chromosome DNAme distributions were altered before and after ComBat correction in mixed-sex and sex-stratified cohorts.

We applied ComBat to adjust for the cohort of origin batch variable (EPIC, NHBC, or RICHS), see Table 1, in: (i) the full 634-sample cohort, (ii) sex-stratified XX female-only (*n* = 309) and XY male-only (*n* = 325) datasets, and (iii) a randomly stratified cohort that was balanced by sex (*n* = 322, *n* = 312). We confirmed that the cohort of origin variable was balanced across all dataset splits (sex-stratified Fisher’s exact test p = 0.78, randomly stratified dataset Fisher’s exact test p= 0.87). The randomly stratified cohort served as a negative control to assess whether effects on DNAme distributions before versus after batch correction were due to halving the dataset during sex-stratification (dataset size) rather than the sex-stratification itself. Spearman’s rho and the RMSE were calculated for the beta values of each sample before versus after batch correction.

The beta values at sex chromosomal and autosomal loci after Combat adjustment were very similar to the pre-ComBat beta values for the same chromosomes, indicated by high average Spearman’s rho and RMSE values. The similarity of the data pre- and post-ComBat dataset held across all cohort splits: i.e., the full cohort, sex-stratified, or randomly-stratified cohorts for the X or Y chromosome in either sex; see Additional file 1: Table S5 and Figures S5 and S6. These results suggest that ComBat adjustment for a sex-balanced batch variable can be performed without sex-stratifying the data while preserving the distributions of X and Y chromosome DNAme data.

### Analytical methods for X and Y chromosome data should vary based on research question

DNAme data analysis typically takes the form of testing for differences in mean DNAme values associated with a phenotype of interest. However, given the sex differences in DNAme associated with XCI and sex chromosome complement, it is not biologically meaningful to directly compare X chromosome DNAme array data between male and female samples. Additionally, Y chromosome DNAme data should only be analyzed in samples possessing a Y chromosome [33].

Recently, a classification scheme was proposed by Beltz et al. [60] to guide analysts in structuring studies of biological sex or sex differences; their recommendations are based on the underlying structures of the data being analyzed [60]. Under this scheme, DNAme patterns on the X and Y chromosome would be characterized as a major qualitative sex difference, and consequently, only within-sex comparisons (i.e., sex-stratified analyses) are biologically valid for X and Y DNAme data. In practice, this means that samples of the same sex can validly be compared across disease status, or with respect to a phenotype of interest, see Table 5. In addition, the involvement of DNAme in the process of XCI allows for a second type of analysis: evaluating the extent of DNAme-associated silencing by XCI in XX female samples. For examples of XCI-based analyses, see [22, 43].

**Table 5:** Statistical comparisons that can be made with X and Y chromosome DNAme array data

Study design	Method
Case vs. control	Sex-stratified (e.g., X chromosome DNAme in XX female case versus unaffected XX female controls)
X-chromosome inactivation	Use average CpG island promoter DNAme to evaluate whether X linked genes escape or are subject to XCI, as demonstrated in [22, 43]

### Application of revised processing workflow enables investigation of X and Y chromosome DNAme alterations associated with acute chorioamnionitis

**Fig. 3:**
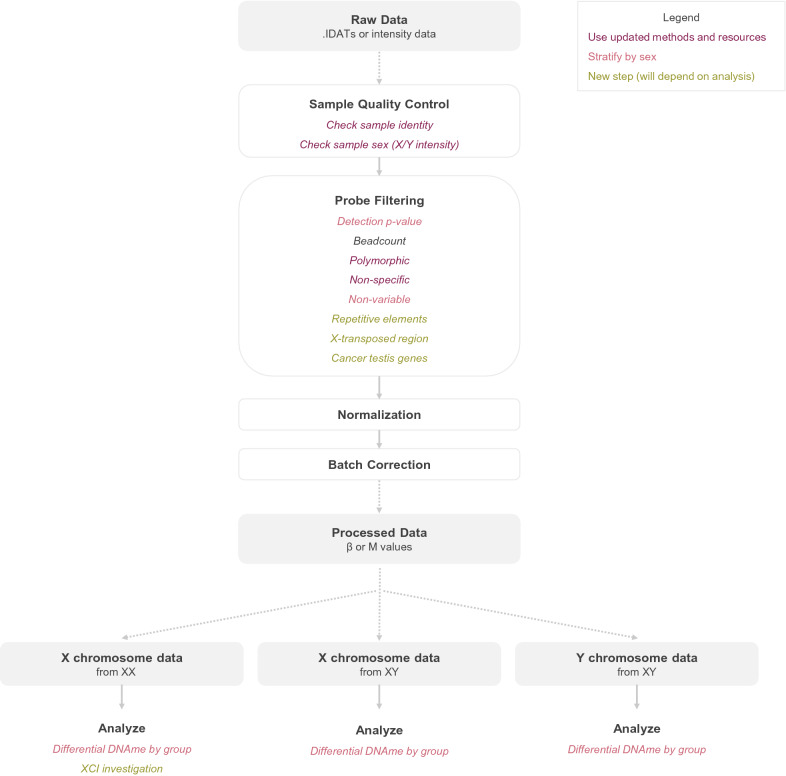
Suggested pipeline for processing and analysis of X and Y chromosome DNAme array data. Overview of all stages of processing from raw data through to processed *β* or *M* values. Colors are used to indicate where updated methods should be used for best results (dark red), which steps need to be stratified by sex (light red), and where new processing/analysis steps are proposed to enable informative sex chromosome investigations (yellow)

For practical application of our recommended modifications to DNAme processing pipelines, we have developed a straightforward R workflow which enables the appropriate integration of X and Y chromosome data in processing mixed-sex DNAme cohorts; see Fig. 3 and available code at https://github.com/amy-inkster/XY_Processing_Analysis (Additional file 4).

**Fig. 4:**
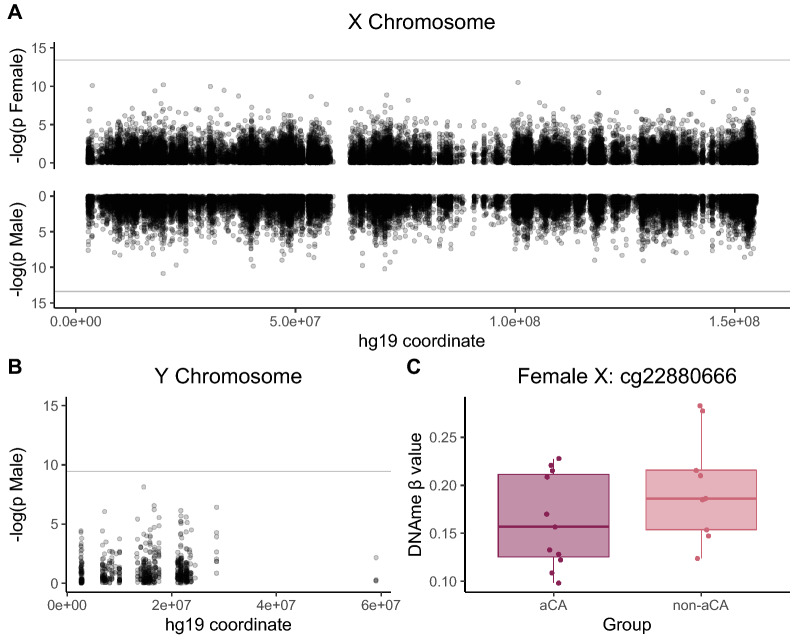
Investigating differential DNAme on the X and Y chromosome by acute chorioamnionitis status. **A** Manhattan plot of X chromosome CpGs tested for DNAme differences between acute chorioamnionitis and non-acute chorioamnionitis samples, with XX samples shown on top row and XY samples on bottom row. The horizontal grey intercept indicates FDR < 0.05, chrX hg19 coordinates are shown along the X axis. **B** Manhattan plot of Y chromosome CpGs tested for DNAme differences between acute chorioamnionitis and non-acute chorioamnionitis XY male placental samples. The horizontal grey intercept indicates FDR < 0.05, chrY hg19 coordinates are shown along the X axis. **C** Distribution of DNAme *β* values for acute chorioamnionitis-affected (aCA) versus unaffected (non-aCA) placental samples at the *NKAP* X chromosomal locus (cg22880666). At FDR < 0.05, this CpG was significantly differentially methylated by aCA status

As a proof-of-principle, we applied our workflow to re-analyze a previously published placental DNAme dataset, see Table 6, to test for differential X and Y chromosome DNAme in acute chorioamnionitis (aCA), an inflammatory condition characterized by infiltration of maternal neutrophils across the chorioamniotic membranes [61]. Our original analysis of this 44-sample cohort in Konwar et al. [61] focused on autosomal DNAme alterations [61]. Here, DNAme associated with aCA status was evaluated on the X and Y chromosome using sex-stratified linear models, adjusting for gestational age at birth. After multiple test correction, no CpGs were differentially methylated at FDR < 0.05 on the male X or Y with respect to aCA status. One CpG in the gene body of *NKAP* was differentially methylated with aCA on the female X (Δβ_non-aCA – aCA_ = 0.048, FDR < 0.05); see Fig. 4. This gene encodes an activating protein for nuclear factor kappa beta (NF-кβ), which is a regulator of the innate immune response [62, 63].

**Table 6:** Acute chorioamnionitis dataset demographics

	Female (XX)	Male (XY)	p-value*
*N*	20	24	
aCA diagnosis [*n* (%)]	9 (45.0%)	13 (54.2%)	
Gestational age [weeks, mean (SD)]	31.00 (2.13)	31.67 (2.82)	0.390

Demographics of 44 chorionic villus samples from GSE115508 used for proof-of-principle analysis

^*^p-values are from Fisher’s exact test for categorical variables and t-tests for continuous variables

## Discussion

A growing body of evidence suggests that autosomal DNAme sex differences are abundant in human tissues [9, 10, 14, 64, 65]. Unfortunately, few studies to date have investigated DNAme profiles of the X or Y chromosome, especially in large population-based cohorts [17–20]. This exclusion is an analytical choice, as data from all chromosomes including the X and Y are de facto collected by all genome-wide assays, including DNAme array, reduced-representation bisulfite sequencing, and whole-genome bisulfite sequencing. There is ample evidence from animal models that the sex chromosomes specifically, as opposed to gonadal hormones, drive a large amount of variability in phenotypic sex differences in a variety of tissues and disease contexts [66, 67], underscoring the importance of including the X and Y chromosomes in all classes of biomedical ‘omics studies.

This work demonstrates how the X and Y chromosome can be incorporated into DNAme array studies going forward. An important finding was the requirement to assess probe failure by high detection p-value thresholding only in XY samples for the Y chromosome. If this step is conducted in all samples of a mixed-sex cohort simultaneously, the majority of Y chromosome probes on the 450K array fail this step. However, when assessed only in male (XY) samples, only 3 out of 416 Y chromosome probes failed, allowing many more Y chromosome probes to be retained for downstream analysis. Several automated methods exist to simplify detection p-value calling and removal into single R functions: these methods in most cases would excessively exclude Y chromosome probes if applied to mixed sex cohorts. As such, detection p-value filtering should be undertaken with caution, particularly if using single-step functions during processing.

We also found that a large degree of variability in resources indexing non-specific and polymorphic (SNP) probes, with respect to their coverage of the X and Y chromosomes. For non-specific probes lists, a recent annotation based on empirical evidence has increased the stringency for indexing cross-hybridization, and recommended more probes be excluded than previously indexed [36]. The authors of this resource, Zhou et al. [36], found evidence for non-specificity with as little as a 13-nucleotide homology. Accordingly, we strongly recommend selecting and using empirically validated non-specific probe lists such as this [36].

Regarding polymorphic probes, variable X and Y chromosome coverage appeared to be mainly mediated by the age of the respective annotations or probe exclusion lists. Though using up-to-date polymorphic probe resources improves coverage of the X and Y chromosome, this is not an ideal solution. An improved approach is offered by the *UMtools* package, which was developed to capitalize on patterns in the underlying fluorescence intensity data reported by Illumina’s DNAme probes [33]. The data-driven tools available in this package enable discernment between genetic interference with DNAme probes and likely true genetically-influenced DNAme (i.e., methylation quantitative trait loci). Further, identifying genetic interference within the dataset itself enables population-specific calling of polymorphic probes, offering a substantial advantage over previous probe exclusion lists when the sample size is large, or includes populations that have been historically underrepresented in polymorphism databases [33].

Normalization and ComBat batch correction were evaluated in this cohort to assess any potential unsuitability of applying these techniques to X and Y chromosome DNAme distributions. We found no evidence for significant alteration of the X or Y chromosome DNAme distributions before versus after ComBat treatment in a mixed-sex cohort, which is reassuring as several reports have highlighted the potential for ComBat to introduce false positives when applied other than as intended by the package authors [57–59]. This is particularly relevant when studying the X and Y chromosome, as sex must be considered as a potential confounder, and should therefore be balanced by all other variables of interest for appropriate application of ComBat.

We similarly found no large differences between raw and normalized DNAme data distributions for the X and Y chromosome. These findings suggest similarity between algorithms, but are independent of consistent effects that may be induced by all methods at autosomal or sex chromosome loci when retaining the X and Y chromosome in a dataset during normalization or ComBat, which is an active area of research [51]. Adjusted methods are now available for both dasen and functional normalization that normalize the sex chromosomes and autosomes independently and with a revised technique, intended to prevent subtle systematic alteration of data induced at both autosomal and X chromosome loci during normalization of mixed-sex cohorts, particularly when sample size is small [51]. These adjusted methods should be considered for use in all studies of the X or Y chromosome, and in studies of autosomal sex-associated DNAme. However, in cases where raw data are not available and choice of normalization algorithm is thus restricted to older methods that can be applied without IDAT files or fluorescent intensities, such as BMIQ, our results suggest limited between-algorithm differences in effect on X and Y DNAme distributions.

The sex-differential DNAme distributions observed on the X and Y chromosome restrict the number of statistically valid analytical approaches that can be conducted at these loci to sex-stratified and XCI-based studies. These analytical approaches would apply both to the analysis of single CpG sites to identify differentially methylated CpGs (DMCs), and to the analysis of differentially methylated regions (DMRs) that comprise multiple CpGs. When regions are defined prior to statistical testing such as in the discovery of co-methylated regions (CMRs) [68], it could be interesting to identify CMRs in a mixed sex cohort, to enable the study of functional units of DNAme that behave similarly in both sexes with respect to a phenotype of interest, perhaps even if the analysis will be sex-stratified.

The ability to analyze X and Y chromosome DNAme data could prompt extensive reanalysis of existing datasets, and opens a variety of novel research avenues for future investigation. In particular, the X and Y should be considered in analyses of any conditions which exhibit sex-differences, such as many immune-related processes [66, 69, 70]. Additional investigations into genetically-mediated DNAme on the sex chromosomes may also prove interesting. For example, very few SNPs on the Y chromosome are proximal to CpGs covered by the 450K or EPIC arrays [36, 37], though literature indicates that Y chromosome DNAme may be strongly genetically mediated in blood, regardless of SNP proximity [17]. Future research should also investigate the extent to which X chromosome DNAme profiles are affected by within-tissue heterogeneous XCI versus skewed XCI (preferential inactivation of one parental allele), if at all. Should DNAme patterns at certain loci vary with degree of skewing, this would have implications for studies of clonally derived tissues, and tissues for which XCI skewing naturally increases with age, including blood and buccal swab [71–73].

## Conclusions

In summary, our study highlights the usability of X and Y chromosome DNAme array data. Importantly, with careful consideration of sample sex during the probe filtering and analysis stages, most Illumina DNAme array datasets will be suitable for sex chromosome analysis. This is a young area of research that will continue to evolve as new discoveries are made. We hope that this method will facilitate the deeper investigation of sex chromosome DNAme profiles in human phenotypes and diseases, particularly in those contexts in which sex differences are abundant.

## Methods

All analyses were performed in R version 4.1.2 [74]. The color-blind friendly palettes used in all plots are from Tol 2021 [75].

### Cohort assembly

Illumina DNAme array data were assembled from 949 human placental samples with publicly available Illumina Infinium HumanMethylation450 array data (GSE71678, GSE74738 GSE75248, GSE98224, GSE100197, GSE108567). We prioritized selection of our own datasets, where samples were taken from the fetal facing side of the placenta, avoiding any potentially contaminating maternal decidua tissue, and washed several times to eliminate the potential for contaminating maternal blood. Any suspected contamination in public datasets was identified and excluded during subsequent quality control steps. Inclusion criteria were: data available in IDAT format, no preeclampsia, birth  > 37 weeks of gestation. After applying inclusion criteria, 711 samples remained for analysis.

### Quality control and probe filtering

Sample sex was assessed using methods from the *ewastools* R package [76]. Sample contamination was identified using the *ewastools* call_genotypes() function, and samples with a high probability of contamination based on a snp_outliers() value of > −4 were removed, as recommended in the original publication [76]. Following quality control, 634 samples remained for analysis. Each step of probe filtering (rs, detection p > 0.01, bead count < 3, polymorphic, non-specific) was evaluated in the full cohort, as well as in sex-stratified cohort halves (*m* = 325 XY males, *n* = 309 XX females). Probes were removed in all cases if they reported a detection p > 0.01 in > 1% of samples, or a bead count < 3 in > 1% of samples. To remove non-variable probes in a statistically valid way, probe variability was first indexed within the test dataset, and then overlapped with a tissue-specific probe exclusion list [35]. Here, we defined non-variable probes as in Edgar et al. [35] as probes with less than a 0.05 range in beta values between the 10th–90th centile in our cohort, and removed these probes from the dataset if they were also reported in the independent list of placenta non-variable probes from Dieckmann et al. [35, 77].

### Normalization

Seven normalization procedures were selected based on consistent demonstration of their high performance in the literature [48, 78–80]: functional, functional + noob, beta-mixture quantile (BMIQ), BMIQ + noob, dasen, dasen + noob, and noob normalization alone. We computed the Spearman correlation coefficient (rho) and root-mean square errors (RMSE) of each sample’s raw versus normalized data. Literature precedent exists for evaluating normalization techniques with RMSE values exists, including for the sex chromosomes [51, 81]. As a supplement we have also included Spearman correlation coefficients, as this metric has been previously utilized for comparing overall sample similarity in DNAme arrays, as well as for monitoring normalization and batch correction effects on DNAme data in technical replicate samples [82, 83].

### Batch correction

To assess bias introduction during batch correction with *sva* Combat [55], batch correction was evaluated using three cohort splits: (i) the full 634-sample cohort, (ii) sex-stratified XX female-only (*n* = 309) and XY male-only (*n* = 325) datasets, and (iii) a randomly stratified cohort that was balanced by sex (*n* = 322 (49% female), *n* = 312 (49% female)). The randomly stratified cohort served as a negative control to assess whether differences observed after batch correction applied to the sex-stratified and full datasets were due to dataset size rather than the sex-stratification itself. The cohort of origin was provided to ComBat as the batch variable (RICHS, NHBC, and EPIC, see Table 1), no outcomes of interest were provided via linear model to the ComBat function. Batch correction was evaluated by computing the Spearman correlation coefficient and RMSE for each sample before versus after ComBat.

### Application of the method to an external dataset

As an application of our processing method, we downloaded Illumina Infinium MethylationEPIC DNAme data for 79 chorionic villus, amnion, and chorion samples from the GSE115508 dataset profiling acute chorioamnionitis. After excluding amnion and chorion tissue samples and removing 4 technical replicates, 44 chorionic villus samples remained, all of which were confirmed to be genetically distinct, and which matched annotated sample sex based on X and Y chromosome fluorescence intensity data. Data were noob normalized, and non-specific and polymorphic probes were removed using the Zhou et al. annotation (*X* chromosome = 2,223 probes removed; *Y* chromosome = 217 probes removed) [36]. Probes with a bead count < 3 in > 5% of samples were excluded (17 CpGs eligible to remove from the X chromosome, 1 from the Y), while detection p-value calling was performed stratified by sex to exclude probes with a detection p > 0.01 in > 5% of samples (556 CpGs eligible to remove from the X chromosome, 0 from the Y). After filtering, 16,353 X chromosome and 320 Y chromosome CpGs remained for analysis. X and Y chromosome DNAme data were retained and converted to M values for all statistical analysis, which consisted of linear modelling in sex-stratified analyses, adjusting for gestational age at birth.

## Supplementary Information

**Additional file 1: Table S1.** Sex-imputation packages and functions available in R. **Table S2.** Provided as separate .xlsx file for download. **Table S3.** Provided as separate .xlsx file for download. **Table S4.** RMSE evaluation of normalization methods. **Table S5.** Evaluation of stratification during ComBat batch correction. **Figure S1.** Sample sex assessments with X and Y chromosome fluorescence intensity data. **Figure S2.** DNA methylation profiles of the X and Y chromosome in placenta and blood (from GSE84727). **Figure S3. **Overlap of non-specific probe resources. **Figure S4**. Overlap of polymorphic probe resources. **Figure S5.** Evaluation of normalization and batch correction methods. **Figure S6. **Principal components analysis before and after ComBat batch correction.

**Additional file 2: Table S2.** Extended XY probe annotation for Illumina 450K array.

**Additional file 3: Table S3.** Extended XY probe annotation for Illumina EPIC array.

**Additional file 4.** R Script for acute chorioamnionitis analysis using GSE115508.

## Data Availability

Code used for processing and analysis of X and Y chromosome DNAme data is available at https://github.com/amy-inkster/XY_Processing_Analysis. All datasets used are publicly available via the Gene Expression Omnibus repository at the indicated accession numbers (https://www.ncbi.nlm.nih.gov/geo/).
