# Supplementary material for: Who’s afraid of the X? Incorporating the X and Y chromosomes into the analysis of DNA methylation array data
Source: Epigenetics Chromatin. 2023 Jan 7;16:1. doi: 10.1186/s13072-022-00477-0 (PMC9825011; doi:10.1186/s13072-022-00477-0)
Supplement: Supplementary file 1 — Additional file 1: Table S1. Sex-imputation packages and functions available in R. Table S2. Provided as separate .xlsx file for download. Table S3. Provided as separate .xlsx file for download. Table S4. RMSE evaluation of normalization methods. Table S5. Evaluation of stratification during ComBat batch correction. Figure S1. Sample sex assessments with X and Y chromosome fluorescence intensity data. Figure S2. DNA methylation profiles of the X and Y chromosome in placenta and blood (from GSE84727). Figure S3. Overlap of non-specific probe resources. Figure S4. Overlap of polymorphic probe resources. Figure S5. Evaluation of normalization and batch correction methods. Figure S6. Principal components analysis before and after ComBat batch correction. [file 13072_2022_477_MOESM1_ESM.pdf]

Supplementary Table 1. Sex-imputation packages and functions available in R.

R Package	Function Name	Data requirement	Method	Aneuploidy detection?
`minfi`	`getSex()`	IDAT files	Median X and Y fluorescence intensity inform sex.<br><br>Note: will not run in single-sex datasets	No
`ewastools`	`check_sex()`	IDAT files	Mean total X and Y fluorescence intensity normalized per-sample to mean total autosomal intensity	No*
`Sesame`	`inferSex(); inferSexKaryotypes()`	IDAT files	Both the fraction of X chromosome CpGs with DNAme β values between 0.3-0.7 (indicating XCI) and mean X and Y fluorescence intensity normalized to per-sample mean autosomal intensity considered.	Yes
`sEst`	`estimateSex()`	Beta values	Distribution of X and Y chromosome beta values used to inform sex	No**
`wateRmelon`	`predictSex()`	Beta values	Principal components analysis on X and Y chromosome beta values	Yes
`RnBeads`	`rnb.execute.gender.prediction()`	IDAT files or raw data as RnBeadRawSet	Ratio of average X and Y intensity to average autosomal intensity	No

**ewastools::check_sex() logic and output can be used to manually identify aneuploidy but does not automatically estimate this.*
***sEst::estimateSex() accurately classifies most aneuploid samples as "N" or null sex, but does not report the estimated aneuploid karyotype.*

Supplementary Table 2 - provided as separate .xlsx file for download.

Supplementary Table 3 - provided as separate .xlsx file for download.

Supplementary Table 4. RMSE evaluation of normalization methods. Mean Spearman rho and root-mean square error values for all samples from the different normalization algorithms. RMSE refers to root mean square error, BMIQ refers to beta-mixture quantile normalization.

	Method (ordered by decreasing Spearman rho)	Mean Spearman rho	Method (ordered by increasing RMSE)	Mean RMSE
**Female X**	**Functional**	0.9996	**Functional**	0.023
	**BMIQ**	0.9972	**Dasen**	0.033
	**Dasen**	0.9970	**BMIQ**	0.037
	**Noob**	0.9966	**Noob**	0.042
	**Functional + noob**	0.9964	**Functional + noob**	0.042
	**Dasen + noob**	0.9959	**Dasen + noob**	0.047
	**BMIQ + noob**	0.9959	**BMIQ + noob**	0.059
**Male X**	**Functional**	0.9994	**Functional**	0.019
	**Noob**	0.9931	**Dasen**	0.03
	**Functional + noob**	0.9923	**BMIQ**	0.045
	**Dasen**	0.9900	**Functional + noob**	0.045
	**Dasen + noob**	0.9889	**Noob**	0.047
	**BMIQ**	0.988	**Dasen + noob**	0.048
	**BMIQ + noob**	0.9842	**BMIQ + noob**	0.068
**Male Y**	**Functional**	0.9979	**Dasen**	0.033
	**BMIQ**	0.9964	**Functional**	0.034
	**Noob**	0.9959	**BMQ**	0.042
	**Dasen**	0.9958	**Noob**	0.044
	**Functional + noob**	0.9946	**Dasen + noob**	0.047
	**Dasen + noob**	0.9945	**Functional + noob**	0.048
	**BMIQ + noob**	0.9933	**BMIQ + noob**	0.063

Supplementary Table 5. Evaluation of stratification during ComBat batch correction. RMSE refers to root mean square error. Sex-stratified indicates males (XY) and females (XX) were batch-adjusted separately from each other, random-stratified indicates the dataset was randomly split in half with some samples of both sexes represented in each half.

	**Dataset stratification (ordered by decreasing Spearman rho)**	**Mean Spearman rho**	**Dataset stratification (ordered by increasing RMSE)**	**Mean RMSE**
**Female X**	**Sex-stratified**	0.9967	**No stratification**	0.0231
	**No stratification**	0.9966	**Random-stratified**	0.0233
	**Random-stratified**	0.9965	**Sex-stratified**	0.0235
**Male X**	**No stratification**	0.9981	**Random-stratified**	0.0216
	**Random-stratified**	0.9981	**No stratification**	0.0218
	**Sex-stratified**	0.9978	**Sex-stratified**	0.0225
**Male Y**	**No stratification**	0.9976	**Sex-stratified**	0.0278
	**Random-stratified**	0.9976	**Random-stratified**	0.0231
	**Sex-stratified**	0.9961	**No stratification**	0.0233

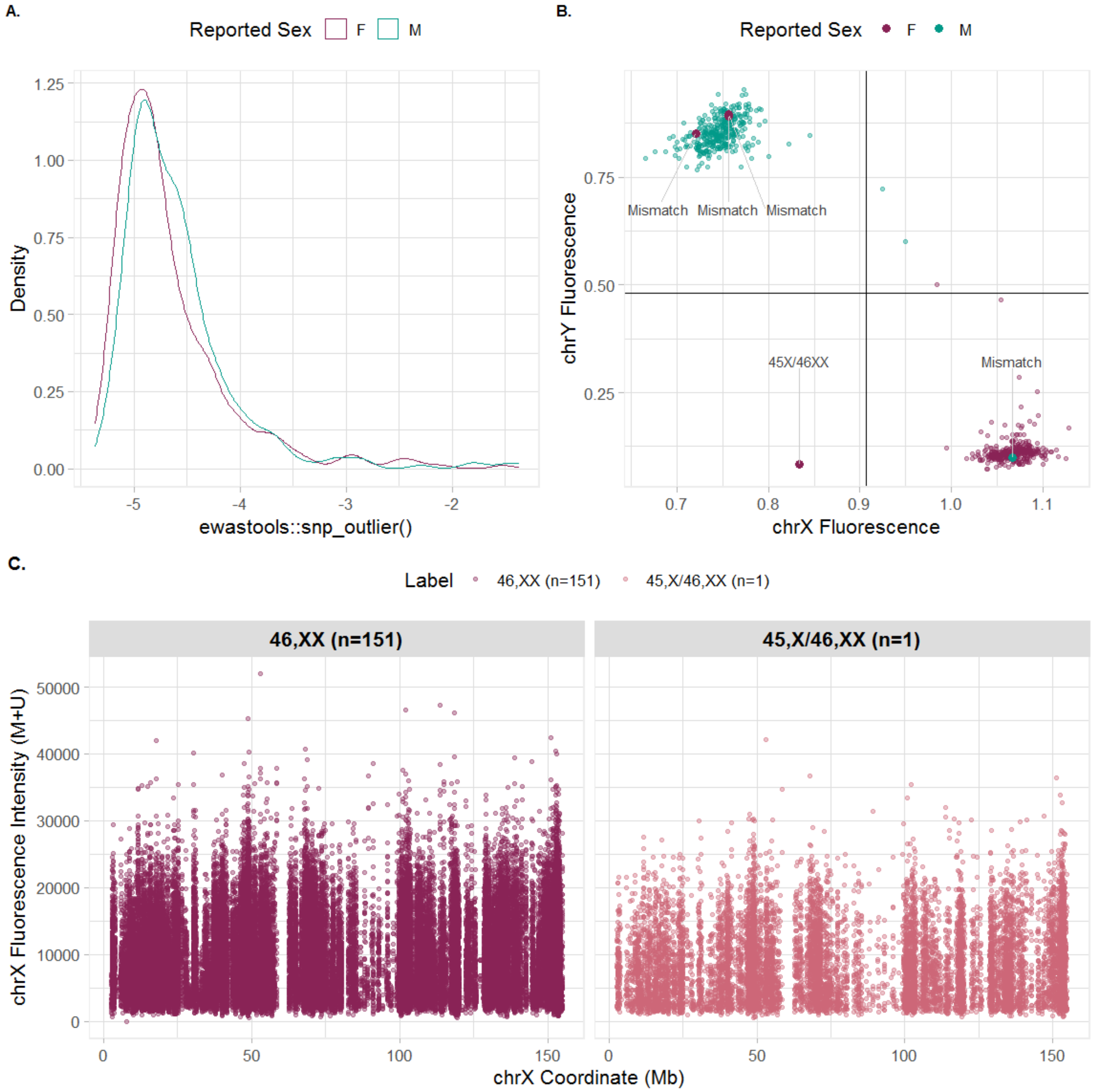

Supplementary Figure 1. Sample sex assessments with X and Y chromosome fluorescence intensity data. (A) Genetic contamination assessment performed with ewastools functions indicated samples with a high probability of contamination (snp_outliers() value > -4). There was no difference in the proportion of male (teal) and female (salmon) samples with high contamination probability. **(B)** Mean X chromosome fluorescence per sample versus mean Y chromosome fluorescence per sample (sum of methylated and unmethylated intensities). Two distinct sample groups are observable, colored by reported sex: reported females in salmon, reported males in teal. **(C)** Total X chromosome fluorescence (sum of methylated and unmethylated intensities), in 151 female samples from GSE71678 (salmon, left) and 1 sample identified to have 45,X/46,XX karyotype (teal, right) from the same GSE71678 dataset, sample ID GSM1843109. Lower total intensity across the entire X chromosome corresponds to less X chromosomal genetic material in this sample. chrX hg19 coordinates are plotted along the X axis.

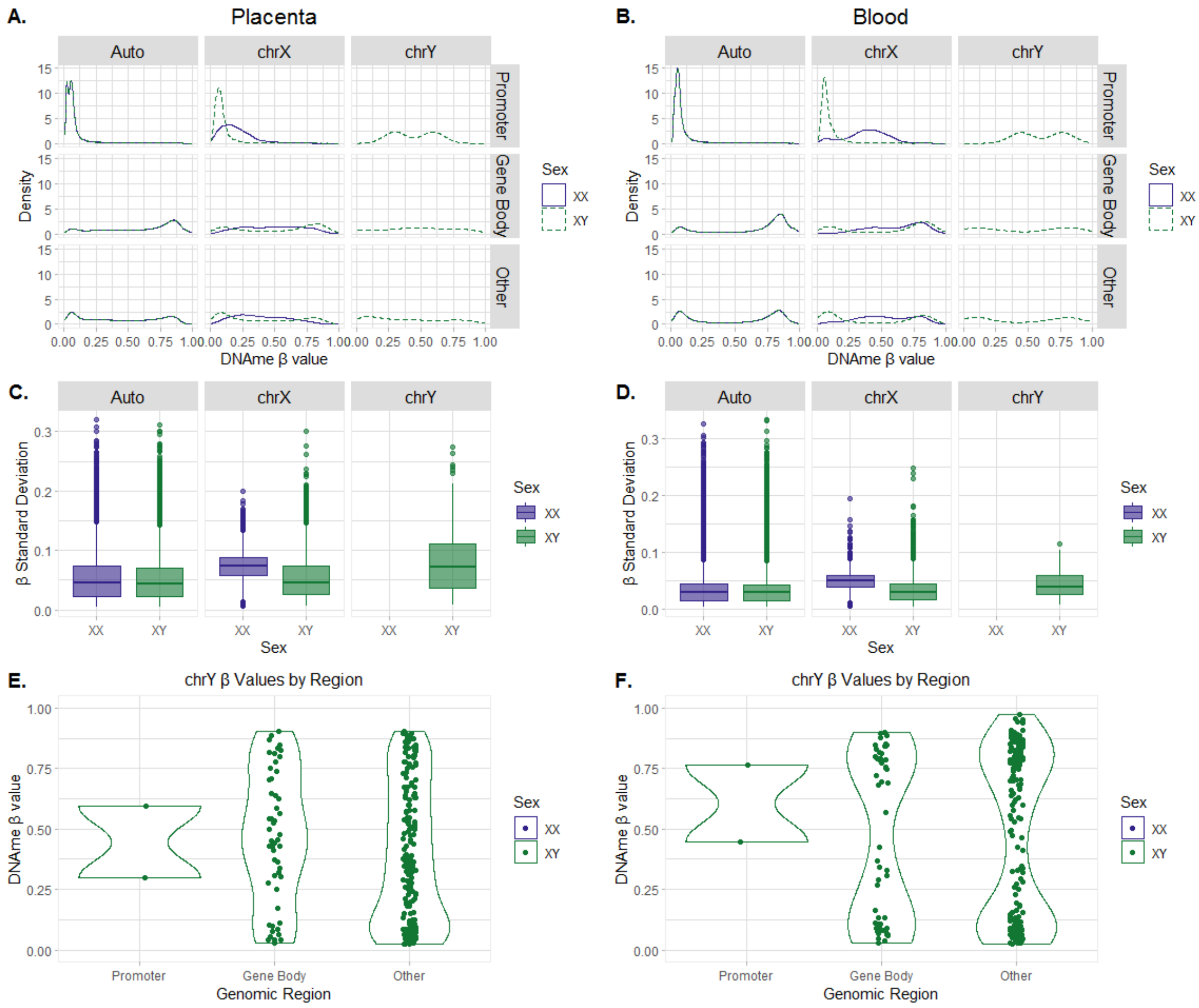

G.

	Autosomal	X Chromosome	Y Chromosome
Promoter	67,922 (0.17)	1,754 (0.19)	2 (0.008)
Gene Body	135,487 (0.34)	2,073 (0.22)	54 (0.22)
Other	193,903 (0.49)	5,573 (0.59)	185 (0.76)

Supplementary Figure 2. DNA methylation profiles of the X and Y chromosome in placenta and blood (from GSE84727). (A) Density plot of raw autosomal, X chromosome, and Y chromosome beta values from placenta. Plots are separated by probe genomic location based on Illumina's UCSC_RefGene_Group category. XX samples beta value densities are in blue, XY samples are shown in dashed green. **(B)** Density plot of raw (no normalization) autosomal, X chromosome, and Y chromosome beta values from blood. Plots are separated by probe genomic location based on Illumina's UCSC_RefGene_Group category. XX samples beta value densities are in blue, XY samples are shown in dashed green. **(C)** Standard deviation of beta values at probes on autosomal, X, and Y chromosomes in placenta. XX samples are in blue, XY samples are in green. Boxplots denote median and the 1st and 3rd quartiles. **(D)** Standard deviation of beta values at probes on autosomal, X, and Y chromosomes in blood. XX samples are in blue, XY samples are in green. Boxplots denote median and the 1st and 3rd quartiles. **(E)** Density plots of placenta DNAme beta values on the Y chromosome separated into functional regions. **(F)** Density plots of blood DNAme beta values on the Y chromosome separated into functional regions.**(G)** Number of probes (after filtering) mapping to functional genomic regions from the autosomes, X, and Y chromosome. Bracketed values indicate the proportion of total filtered probes from that chromosome mapping to the functional region.

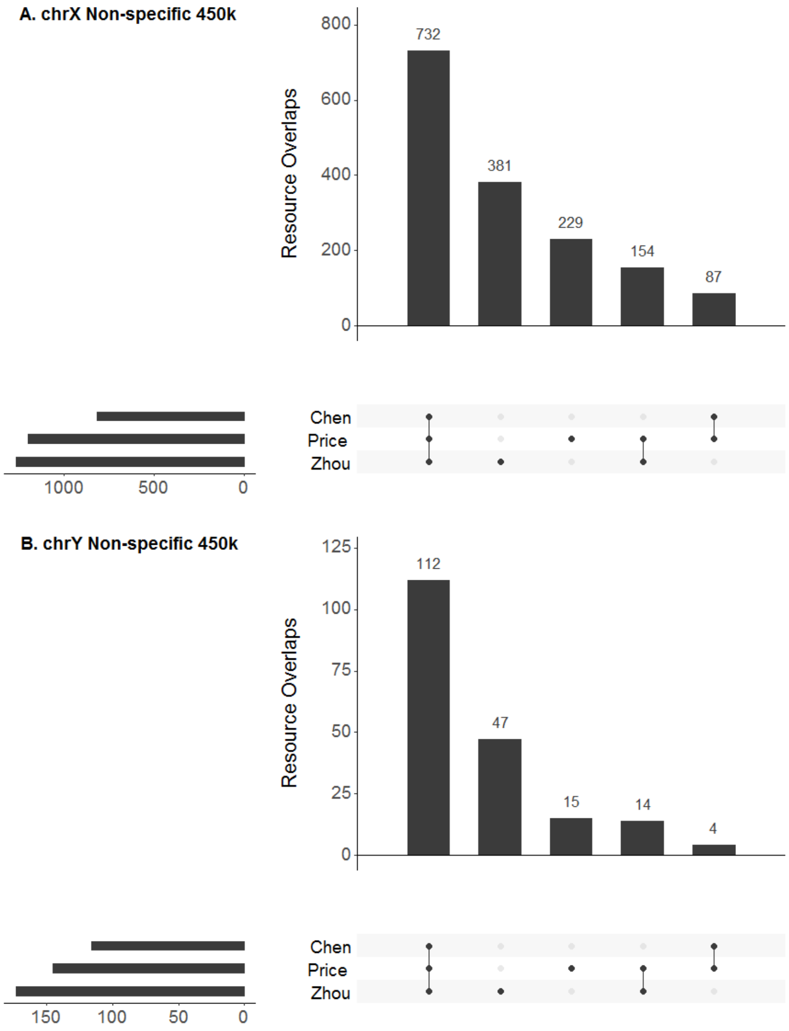

Supplementary Figure 3. Overlap of non-specific probe resources. Upset plots showing the overlap of common 450K non-specific probe exclusion lists. In all plots the probe resources are named by the surname of the first author or the formal name of the tool if applicable. Chen: Chen et al. 2013 [1], Price: Price et al. 2013 [2], Zhou: Zhou et al. 2017 [3]. **(A)** X chromosome coverage in non-specific probe resources. **(B)** Y chromosome coverage in non-specific probe resources.

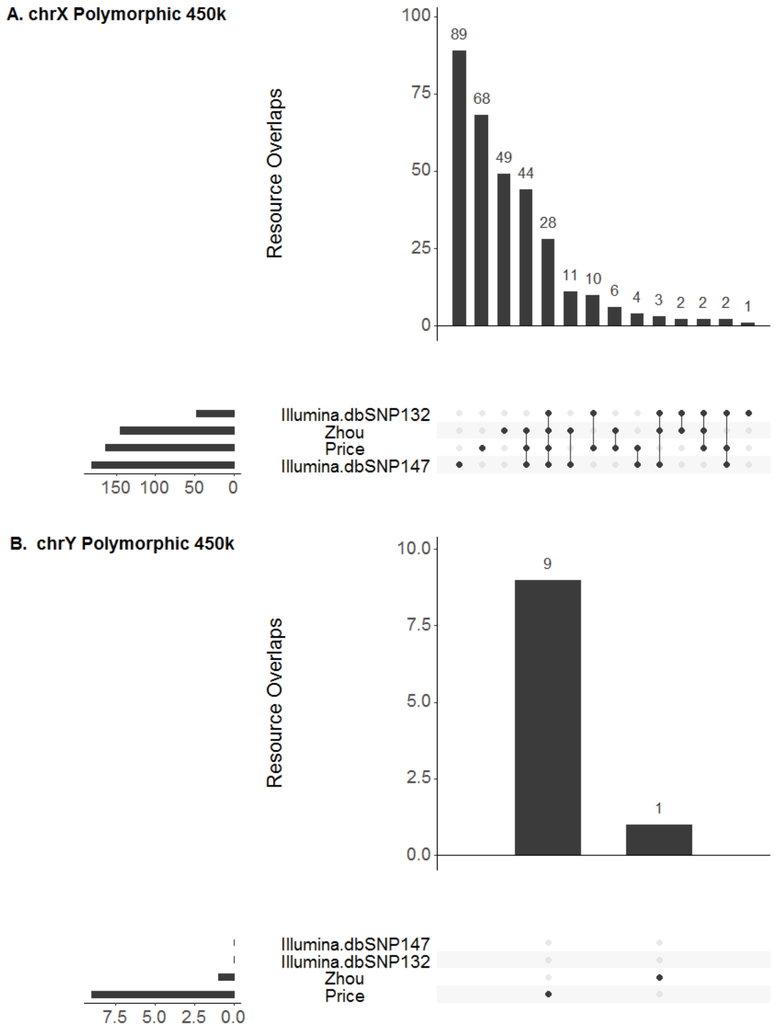

Supplementary Figure 4. Overlap of polymorphic probe resources. Upset plots showing the overlap of common 450K polymorphic probe exclusion lists. In all plots the probe resources are named by the surname of the first author or the formal name of the tool if applicable. Price: Price et al. 2013 [2], Zhou: Zhou et al. 2017 [3].. **(A)** X chromosome coverage in polymorphic probe resources. **(D)** Y chromosome in polymorphic probe resources.

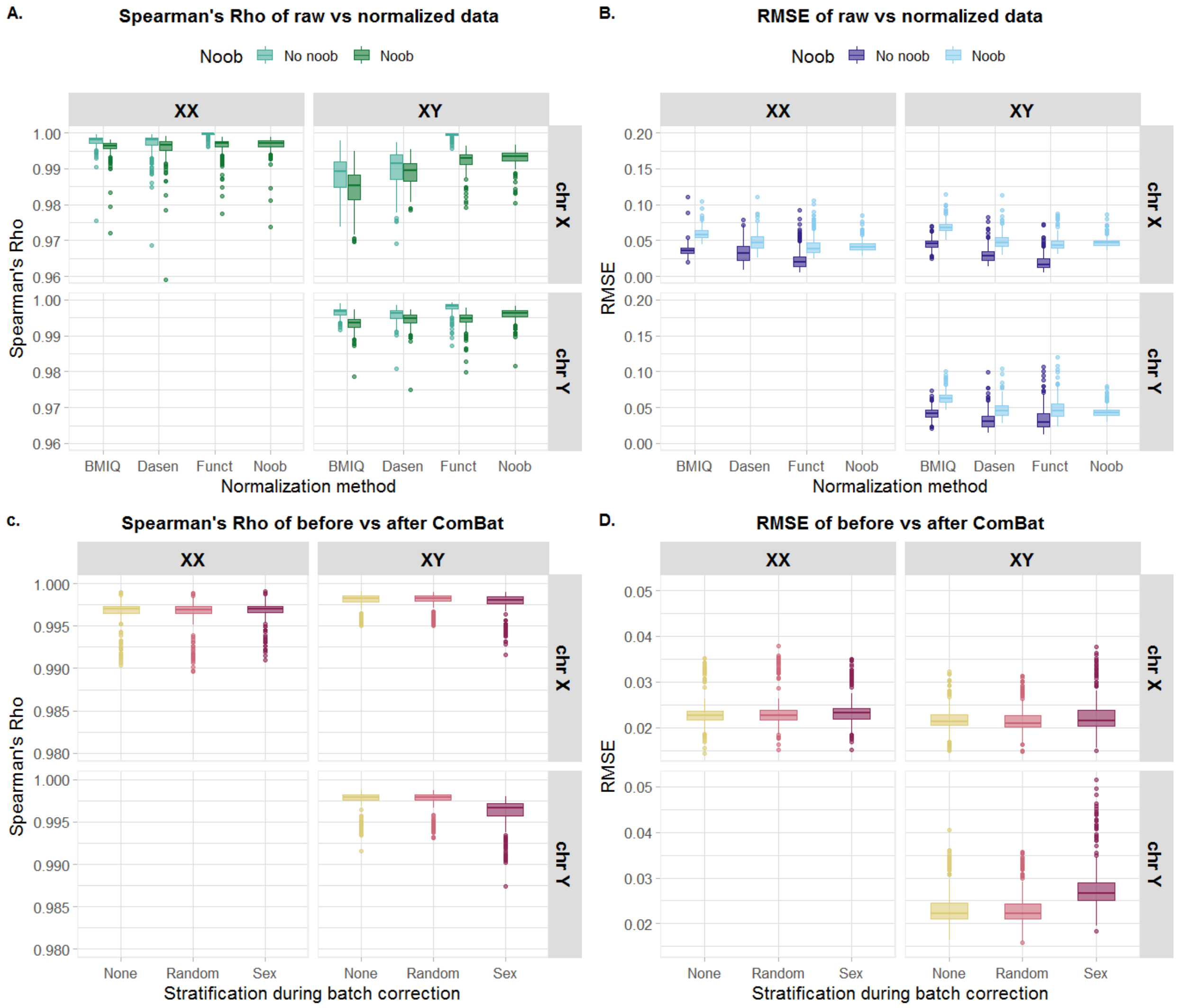

Supplementary Figure 5. Evaluation of normalization and batch correction methods. (A) Spearman correlation (rho) coefficients for all samples before and after normalization with the seven different normalization methods, plotted separated by sex (XX female, XY male) and chromosome (X, Y). "Funct" refers to functional normalization. **(B)** RMSE for all samples before and after normalization for all normalization methods tested, plotted separated by sex (XX female, XY male) and chromosome (X, Y). "Funct" refers to functional normalization. **(C)** Spearman correlation (rho) coefficients for all samples before and after ComBat correction, shown for not stratified ("none"), randomly stratified ("random"), and sex-stratified ("sex") datasets. Correlation results are shown separated by sex (XX female, XY male) and chromosome (X, Y). **(D)** RMSE for all samples before and after ComBat correction, shown for not stratified ("none"), randomly stratified ("random"), and sex-stratified ("sex") datasets and plotted separated by sex (XX female, XY male) and chromosome (X, Y).

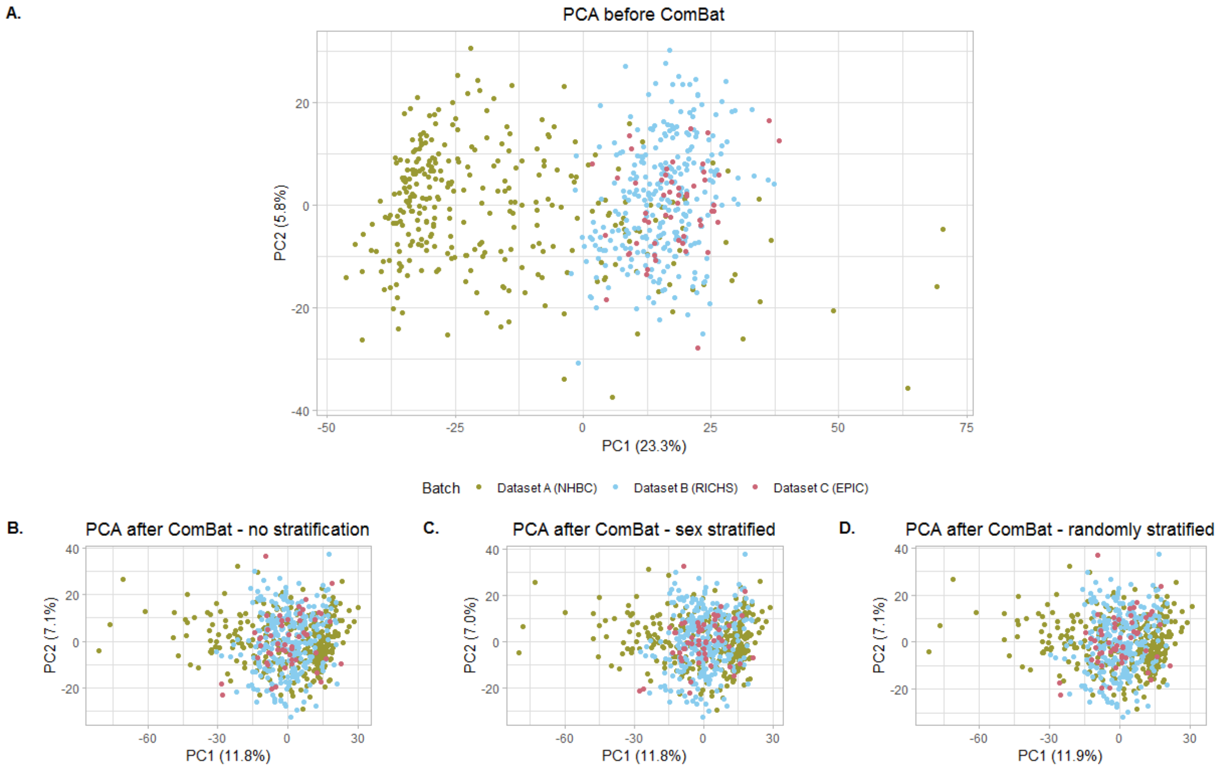

Supplementary Figure 6. Principal components analysis before and after ComBat batch correction. (A) Principal components analysis (PCA) scatterplot of the first principal component (PC1) versus the second principal component (PC2) before batch correction with sva ComBat. **(B)** Scatterplot of PC1 versus PC2 after batch correction with sva ComBat in the full dataset. **(C)** Scatterplot of PC1 versus PC2 after batch correction with sva ComBat run on sex-stratified subsets of the dataset. **(D)** Scatterplot of PC1 versus PC2 after batch correction with sva ComBat randomly stratified subsets of the dataset. For all plots points are colored by dataset, the batch-corrected variable, Dataset A corresponds to the New Hampshire Birth Cohort (NHBC), Dataset B corresponds to the rhode Island Child Health Study cohort (RICHS), and Dataset C corresponds to the Epigenetics in Pregnancy (EPIC) cohort.

Supplementary References

1. Chen Y, Lemire M, Choufani S, Butcher DT, Grafodatskaya D, Zanke BW, et al. Discovery of cross-reactive probes and polymorphic CpGs in the Illumina Infinium HumanMethylation450 microarray. Epigenetics. 2013;8:203–9.

2. Price ME, Cotton AM, Lam LL, Farré P, Emberly E, Brown CJ, et al. Additional annotation enhances potential for biologically-relevant analysis of the Illumina Infinium HumanMethylation450 BeadChip array. Epigenetics & Chromatin. 2013;6:4.

3. Zhou W, Laird PW, Shen H. Comprehensive characterization, annotation and innovative use of Infinium DNA methylation BeadChip probes. Nucleic Acids Res. 2017;45:e22.
