# Supplementary material for: Who’s afraid of the X? Incorporating the X and Y chromosomes into the analysis of DNA methylation array data
Source: Epigenetics Chromatin. 2023 Jan 7;16:1. doi: 10.1186/s13072-022-00477-0 (PMC9825011; doi:10.1186/s13072-022-00477-0)
Supplement: Supplementary file 4 — Additional file 4. R Script for acute chorioamnionitis analysis using GSE115508. [file 13072_2022_477_MOESM4_ESM.pdf]

Sex-stratified linear modelling for differential X and Y chromosome DNAme

Using GSE115508 EPIC data from placentas with and without acute chorioamnionitis

Amy M. Inkster

Contents

0.0 Intro & load packages . . . 1
1.0 DNAme data . . . 2
2.0 Sample Quality Control . . . 3
3.0 Probe filtering . . . 6
4.0 Analysis . . . 8
5.0 Plot hit . . . 10
6.0 Session Info . . . 13

0.0 Intro & load packages

In this script I download the data associated with the acute chorioamnionitis (aCA) dataset (GSE115508) from the Gene Expression Omnibus (GEO) website, process and normalize the X and Y chromosome DNAme data from this cohort in the method of Inkster et al. 2022, and then run a set of linear models to test for differential DNAme associated with aCA status on the X and Y chromosome.

```
library(tidyverse)
library(here)

library(biobroom)
library(ewastools)
library(GEOquery)
library(lumi)
library(minfi)
library(sesameData)
library(wateRmelon)
library(cowplot)
library(IlluminaHumanMethylationEPICanno.ilm10b4.hg19)

# illumina annotation
probeInfo <- as.data.frame(cbind(IlluminaHumanMethylationEPICanno.ilm10b4.hg19::Locations,
                                 IlluminaHumanMethylationEPICanno.ilm10b4.hg19::Other,
                                 IlluminaHumanMethylationEPICanno.ilm10b4.hg19::Manifest))
probeInfo$probeID <- rownames(probeInfo)
chrXprobes <- probeInfo %>% filter(chr == "chrX")
```

```
chrYprobes <- probeInfo %>% filter(chr == "chrY")

# zhou annotation
# https://zwdzwd.github.io/InfiniumAnnotation
# https://zhouserver.research.chop.edu/InfiniumAnnotation/20180909/EPIC/EPIC.hg19.manifest.tsv.gz
zhouAnno <- sesameDataGetAnno("EPIC/EPIC.hg19.manifest.tsv.gz")
```

```
## Retrieving annotation from  https://zhouserver.research.chop.edu/InfiniumAnnotation/current/EPIC/EPI
```

1.0 DNAme data

1.1 Download from GEO

Metadata is stored on the series matrix and raw data in the form of IDAT files can be downloaded from: https://www.ncbi.nlm.nih.gov/geo/query/acc.cgi?acc=GSE115508

Data and metadata parsing code are not shown in this script.

1.2 Load objects

```
pDat <- read.csv(here("GSE115508/GSE115508_pData.csv"))
rgset <- readRDS(here("GSE115508/rgset_ext.rds"))

pDat <- pDat %>% mutate(Sample_ID = paste0(Sentrix_ID, "_", Sentrix_Position)) # to match names of rgse
```

1.3 Subset to chorionic villous samples

In this section we are going to select only data from chorionic villi. This dataset is comprised of matched tissue (chorion, amnion, and chorionic villi) from multiple placentae.

```
pDat <- pDat %>% filter(Tissue %in% "Chorionic villi") # 48 samps
rgset <- rgset[ , pDat$Sample_ID]
all.equal(pDat$Sample_ID, colnames(rgset)) # must be TRUE before proceeding

# now normalize dataset, also get raw betas for sex checks later with minfi
mset <- preprocessFunnorm(rgset, bgCorr = F, dyeCorr = F)
```

```
## [preprocessFunnorm] Mapping to genome

## Loading required package: IlluminaHumanMethylationEPICmanifest

## [preprocessFunnorm] Quantile extraction

## [preprocessFunnorm] Normalization
```

```
mset_raw <- preprocessRaw(rgset)
betas <- minfi::getBeta(mset)
```

```
## [1] TRUE
```

2.0 Sample Quality Control

2.1 Check identity

Use the 56 rs built into the EPIC array to estimate sample genotypes at these loci and compare across all samples.

```
rs <- getSnpBeta(rgset)
genotypes <- call_genotypes(rs)

# label samples with donor ID and number of times present in data genetically
pDat <- pDat %>% mutate(Sample_Donors = enumerate_sample_donors(genotypes))
pDat <- pDat %>% group_by(Sample_Donors) %>% mutate(n_Duplicated = n()) %>% ungroup()

# index samples explicitly labelled "rvc" to easily remove prior to analysis
pDat <-  pDat %>%
  mutate(Replicate = case_when(grepl("_r", Sample_Name) ~ "Replicate"))
```

2.2 Check sex (modified ewastools method)

In this section we evaluate the sex of all samples using a modified version of the ewastools check_sex() function. This code was modified to work with minfi objects to avoid parsing the IDATs into multiple data objects.

```
# ALL CREDIT FOR CODE BELOW IS ATTRIBUTED TO EWASTOOLS PACKAGE AUTHORS (Heiss & Just)
# For ewastools package info please visit https://github.com/hhhh5/ewastools

# Original ewastools paper:
# Heiss, J., Just, A. Identifying mislabeled and contaminated DNA methylation
# microarray data: an extended quality control toolset with examples from GEO.
# Clin Epigenet 10, 73 (2018). https://doi.org/10.1186/s13148-018-0504-1

# function equivalent to ewastools::check_sex(), pull sample XY intensity norm to auto
mset_check_sex = function(mset){

  if(!"tidyverse" %in% installed.packages()) {
    install.packages("tidyverse")
  }


  if(!"BiocManager" %in% installed.packages()) {
    install.packages("BiocManager")
  }

  if(!"minfi" %in% installed.packages()) {
    BiocManager::install("minfi")
  }

  require(tidyverse)
  require(minfi)

  # define M, U, and probe anno objects
  M = minfi::getMeth(mset) %>% as.data.frame()
```

```
  U = minfi::getUnmeth(mset) %>% as.data.frame()
  anno = minfi::getAnnotation(mset) %>% as.data.frame() # annotation with "chr" column, entries chr1 -


  # select allosomal probes
  chrX = dimnames(anno[anno$chr=='chrX',])[[1]]
  chrY = dimnames(anno[anno$chr=='chrY',])[[1]]

  # compute the total intensities
  chrX = colMeans(M[chrX,,drop=FALSE]+U[chrX,,drop=FALSE],na.rm=TRUE)
  chrY = colMeans(M[chrY,,drop=FALSE]+U[chrY,,drop=FALSE],na.rm=TRUE)

  # compute the average total intensity across all autosomal probes
  autosomes = dimnames(anno[!anno$chr %in% c("chrX","chrY"),])[[1]]
  autosomes = colMeans(M[autosomes,,drop=FALSE]+U[autosomes,,drop=FALSE],na.rm=TRUE)

  # normalize total intensities
  chrX_nonorm = chrX
  chrY_nonorm = chrY

  chrX = chrX/autosomes
  chrY = chrY/autosomes

  # define a df with the output
  xy_int = data.frame(Sample_ID = dimnames(mset)[[2]],
                      Array = annotation(mset)[[1]],
                      X = chrX,
                      Y = chrY)

    return((xy_int))
}


# equivalent to ewastools::predict_sex(), calls sex based on XY intensity values
# by calculating robust hodges-lehmann estimator based on known male/female samples
mset_predict_sex = function(sex_int,male,female){

    # compute the robust Hodges-Lehmann estimator for the total intensity for X chr probes
    cutX = outer(sex_int$X[male],sex_int$X[female],"+")
    cutX = median(cutX)/2

    # ... likewise for Y chr probes
    cutY = outer(sex_int$Y[male],sex_int$Y[female],"+")
    cutY = median(cutY)/2

    # Prediction based on in which quadrant (cutX/cutY) samples fall
    DNAme_Sex = rep(NA,times=length(sex_int$X))
    DNAme_Sex[sex_int$X>=cutX & sex_int$Y<=cutY] =  "F"
    DNAme_Sex[sex_int$X<=cutX & sex_int$Y>=cutY] =  "M"
    factor(DNAme_Sex,levels=c("M","F"),labels=c("M","F"))

    sex_preds = cbind(sex_int, DNAme_Sex)
```

```
    results = list("Sex_Preds" = sex_preds, "cutX" = cutX, "cutY" = cutY)

    return(results)

}
```

```
# calculate normalized X and Y fluo intensity per sample & predict sexes
# need logical vectors of male and females in dataset
male <- pDat$Sex == "M"
female <- pDat$Sex == "F"

sex_list <- mset_check_sex(mset_raw)
sex_list <- mset_predict_sex(sex_list, male, female)
head(sex_list$Sex_Preds) # result: sex estimation for each sample with chrX & chrY norm'd fluo intensit[illegible]

# attach X and Y normalized intensities plus predicted sexes to metadata
pDat <- pDat %>% left_join(sex_list$Sex_Preds, by="Sample_ID")

# plot coloring by REPORTED SEX to see how accurate the metadata is
# no samples of concern for sex chr complement
pDat %>%
  ggplot(aes(x=X, y=Y, color=Sex)) +
  geom_point() +
  geom_vline(xintercept = sex_list$cutX) +
  geom_hline(yintercept = sex_list$cutY) +
  labs(title="chrX vs chrY normalized fluorescence intensity",
       color="Reported Sex",
       x="Mean chrX / mean Autosomal Intensity",
       y="Mean chrY / mean Autosomal Intensity") +
  theme_minimal() +
  theme(plot.title = element_text(hjust=0.5),
        plot.subtitle = element_text(hjust=0.5, face="italic"),
        legend.position = "none",
        strip.text = element_text(size=12),
        strip.background = element_rect(fill="#DDDDDD", color= "#DDDDDD"),
        panel.border = element_rect(color= "#EDEDED", fill=NA))
```

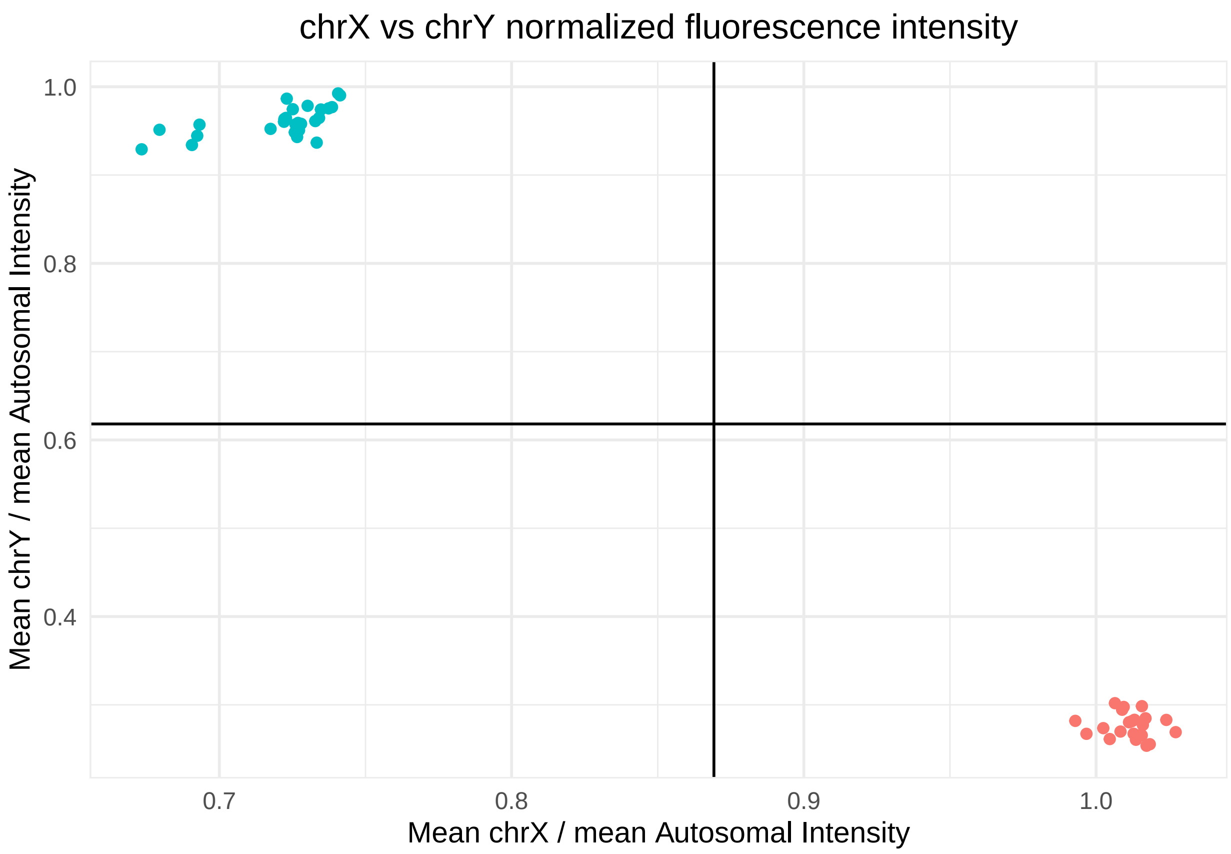


```
##                                Sample_ID                        Array         X
## 200889820007_R01C01 200889820007_R01C01 IlluminaHumanMethylationEPIC 0.7301987
## 200889820007_R02C01 200889820007_R02C01 IlluminaHumanMethylationEPIC 1.0089254
## 200889820007_R03C01 200889820007_R03C01 IlluminaHumanMethylationEPIC 1.0160101
## 200889820007_R04C01 200889820007_R04C01 IlluminaHumanMethylationEPIC 1.0122412
## 200889820007_R05C01 200889820007_R05C01 IlluminaHumanMethylationEPIC 0.7341118
## 200889820007_R06C01 200889820007_R06C01 IlluminaHumanMethylationEPIC 0.7269093
##                             Y DNAme_Sex
## 200889820007_R01C01 0.9784187         M
## 200889820007_R02C01 0.2944260         F
## 200889820007_R03C01 0.2772505         F
## 200889820007_R04C01 0.2814539         F
## 200889820007_R05C01 0.9646551         M
## 200889820007_R06C01 0.9590593         M
```

3.0 Probe filtering

3.1 Detection p value & beadcount

Defining poor quality probes herein those with a detection P value > 0.01 in $>5\%$ of samples. *This is a step that has to be done differently for the Y chromosome - i.e. don't evaluate detP in XX cases!*

Also, remove probes with a beadcount < 3 in $>5\%$ of samples. No need to sex-stratify beadcount failure calling.

```r
# create a 'failed probe' matrix to filter
detp <- minfi::detectionP(rgset)
bc <- beadcount(rgset)

# for female Y chromosome, set detp to 0 (they are not failing so not > 0.05)
detp[rownames(detp) %in% chrYprobes$probeID, female] <- 0

# create a failed probes matrix
 # if detP > 1 OR bc<3 (NA) OR missing value, sum per cell will be > 0
rawbeta <- getBeta(rgset)

fp <- (detp>0.01) + is.na(bc) + is.na(rawbeta)
fp <- fp > 0

fail_probes <- fp[ rowSums(fp) > ncol(fp)*0.05 ,]
dim(fail_probes)

# remove failed probes in > 5% samples
dim(betas) # 865859
betas <- betas[!(rownames(betas) %in% rownames(fail_probes)),]
dim(betas) # 855837

# identify (& remove) samples with > 5% failed probes
table(colSums(fp) > nrow(fp)*0.05) # 0 failed
```

```
## [1] 10022    48
## [1] 865859     48
## [1] 855837     48
##
## FALSE
##    48
```

3.2 Polymorphic & cross-hybridizing probes

No separate treatment required for X and Y data versus autosomes.

```r
# remove probes
zhou_maskgeneral <- zhouAnno %>% filter(MASK_general == TRUE)
dim(zhou_maskgeneral) # 99360 that need to be removed

table(chrXprobes$probeID %in% zhou_maskgeneral$probeID) # 2223 X probes to remove
table(chrYprobes$probeID %in% zhou_maskgeneral$probeID) # 217 Y probes to remove

dim(betas) # 855837
betas <- betas[!(rownames(betas) %in% zhou_maskgeneral$probeID), ]
dim(betas) # 758142
```

```
## [1] 99360    57
##
## FALSE  TRUE
## 16867  2223
##
```

```
## FALSE  TRUE
##   320   217
## [1] 855837     48
## [1] 758142     48
```

3.4 Non-variable probes

In the context of most processing pipelines, now would be the time to remove non-variable probes. For statistical validity with later multiple test correction, we need to do this with reference to an external database or a priori biological knowledge.

Unfortunately, a resource for EPIC placental data that includes the X and Y does not exist yet, so we are limited in our ability to do so. Thus we will leave in the non-variable due to technical limitations, but if we were going to do this Y variability should be assessed in males only! (Call variability and then set female Y values to 0 as we did in the detP section).

4.0 Analysis

4.1 Remove replicates

```
pDat_lm <- pDat %>% filter(is.na(Replicate)) # keep everything that is not a replicate
betas <- betas[,pDat_lm$Sample_ID]

pDat_f <- pDat_lm %>% filter(Sex == "F")
pDat_m <- pDat_lm %>% filter(Sex == "M")

table(pDat_f$Group) # 11aCA vs 9not
table(pDat_m$Group) # 11aCA vs 13not
```

```
##
##     chorioamnionitis non_chorioamnionitis
##                   11                    9
##
##     chorioamnionitis non_chorioamnionitis
##                   11                   13
```

4.2 Select XY probes

We are going to run 3 linear models, Female X, Male X, and Male Y. The data and metadata will need to be separated into these subsets and then subjected to linear modelling.

```
mVals <- beta2m(betas)

m_xf <- mVals[rownames(mVals) %in% chrXprobes$probeID, pDat_f$Sample_ID]
m_xm <- mVals[rownames(mVals) %in% chrXprobes$probeID, pDat_m$Sample_ID]
m_ym <- mVals[rownames(mVals) %in% chrYprobes$probeID, pDat_m$Sample_ID]

dim(m_xf) # 16344 20
dim(m_xm) # 16344 24
dim(m_ym) # 314 24
```

```
## [1] 16344    20
## [1] 16344    24
## [1] 314  24
```

4.3 Linear model

```
# specify design matrix (one for each sex)
mod_f <- model.matrix(~ Group + GA, pDat_f, row.names=T)
mod_m <- model.matrix(~ Group + GA, pDat_m, row.names=T)

# female X
fit_xf <- lmFit(m_xf, mod_f) %>% eBayes()
td_xf <- tidy.MArrayLM(fit_xf) %>%
  dplyr::rename(probeID = gene) %>%
  group_by(term) %>%
  mutate(fdr = p.adjust(p.value, method="fdr")) %>%
  ungroup() %>%
  as.data.frame()
```

```
## Warning: `tbl_df()` was deprecated in dplyr 1.0.0.
## Please use `tibble::as_tibble()` instead.
## This warning is displayed once every 8 hours.
## Call `lifecycle::last_lifecycle_warnings()` to see where this warning was generated.
```

```
td_xf %>% filter(fdr < 0.05) # one hit

# male X
fit_xm <- lmFit(m_xm, mod_m) %>% eBayes()
td_xm <- tidy.MArrayLM(fit_xm) %>%
  dplyr::rename(probeID = gene) %>%
  group_by(term) %>%
  mutate(fdr = p.adjust(p.value, method="fdr")) %>%
  ungroup() %>%
  as.data.frame()

td_xm %>% filter(fdr < 0.05) # 0


# male Y
fit_ym <- lmFit(m_ym, mod_m) %>% eBayes()
td_ym <- tidy.MArrayLM(fit_ym) %>%
  dplyr::rename(probeID = gene) %>%
  group_by(term) %>%
  mutate(fdr = p.adjust(p.value, method="fdr")) %>%
  ungroup() %>%
  as.data.frame()

td_ym %>% filter(fdr < 0.05) # 0
```

```
##      probeID term   estimate statistic      p.value      lod         fdr
## 1 cg22880666   GA -0.2137177 -7.321073 2.390098e-07 5.947596 0.003906377
```

```
## [1] probeID   term      estimate  statistic p.value   lod       fdr
## <0 rows> (or 0-length row.names)
## [1] probeID   term      estimate  statistic p.value   lod       fdr
## <0 rows> (or 0-length row.names)
```

5.0 Plot hit

One CpG was differentially methylated in females on the X chromosome by aCA status. Boxplot to visualize this below, this CpG is in the gene body of the NKAP gene (NFkB activating protein), and is more highly methylated in non-chorioamnionitis placentae.

5.1 Boxplot

```
td_xf %>% filter(fdr < 0.05)

cg22880666 <- betas[rownames(betas) %in% c("cg22880666"), pDat_f$Sample_ID, drop=F] %>%
  t() %>%
  as.data.frame() %>%
  rownames_to_column(., var="Sample_ID") %>%
  left_join(pDat_f)
```

```
## Joining, by = "Sample_ID"
```

```
cg22880666 %>%
  group_by(Group) %>%
  summarize(mean=mean(cg22880666))

plot_xHit <- cg22880666 %>%
  mutate(Group = case_when(Group == "chorioamnionitis" ~ "aCA",
                           Group == "non_chorioamnionitis" ~ "non-aCA")) %>%

  ggplot(aes(x=Group, y=cg22880666, color=Group, fill=Group)) +
  geom_boxplot(outlier.shape=NA, alpha=0.5) +
  geom_point(position=position_jitter(width=0.05)) + # position_jitterdodge(dodge.width = 0.2)) +
  ggtitle("Female X: cg22880666") +
  ylab("DNAme \u03b2 value") +
  theme_classic(base_size=16) +

  theme(plot.title = element_text(hjust=0.5),
        plot.subtitle = element_text(hjust=0.5, face="italic"),
        legend.position = "none",
        strip.text = element_text(face="bold", size=12),
        panel.grid = element_blank()) +

  scale_fill_manual(values=c("#882255", "#cc6677")) +
  scale_color_manual(values=c("#882255", "#cc6677"))


chrXprobes %>% filter(probeID %in% c("cg22880666"))
```

```
##      probeID term   estimate statistic      p.value      lod         fdr
## 1 cg22880666   GA -0.2137177 -7.321073 2.390098e-07 5.947596 0.003906377
## # A tibble: 2 x 2
##   Group                  mean
##   <chr>                 <dbl>
## 1 chorioamnionitis      0.163
## 2 non_chorioamnionitis 0.198
##             chr       pos strand
## cg22880666 chrX 119076813      -
##
## cg22880666 CAATGGTTGAGCGCCTATAAAGTACCAGGCACTTAGAATTCTGTGATCAGAATTCTCCTC[CG]TTCACTGGCTCCTCCTTTCCATGAG
##                                                                   SourceSeq UCSC_RefGene_Name
## cg22880666 CGGAGGAGAATTCTGATCACAGAATTCTAAGTGCCTGGTACTTTATAGGC              NKAP
##            UCSC_RefGene_Accession UCSC_RefGene_Group Phantom4_Enhancers
## cg22880666              NM_024528               Body
##                  Phantom5_Enhancers DMR X450k_Enhancer HMM_Island
## cg22880666 chrX:119076607-119076607
##            Regulatory_Feature_Name Regulatory_Feature_Group
## cg22880666
##            GencodeBasicV12_NAME GencodeBasicV12_Accession GencodeBasicV12_Group
## cg22880666
##            GencodeCompV12_NAME GencodeCompV12_Accession GencodeCompV12_Group
## cg22880666
##            DNase_Hypersensitivity_NAME DNase_Hypersensitivity_Evidence_Count
## cg22880666    chrX:119076605-119076855                                     3
##            OpenChromatin_NAME OpenChromatin_Evidence_Count TFBS_NAME
## cg22880666
##            TFBS_Evidence_Count Methyl27_Loci Methyl450_Loci Random_Loci
## cg22880666                                             TRUE
##                  Name AddressA AddressB
## cg22880666 cg22880666 64724588
##                                                                   ProbeSeqA ProbeSeqB Type
## cg22880666 CRCCTATAAAATACCAAACACTTAAAATTCTATAATCAAAATTCTCCTCC             II
##            NextBase Color    probeID
## cg22880666                cg22880666
```

5.2 Manhattan plots

```
# manhattan plot
plot_manF <- td_xf %>%
  left_join(probeInfo %>% dplyr::select(probeID, pos)) %>%
  ggplot(aes(x=pos, y=-log(p.value))) +
  geom_point(alpha=0.2) +
  geom_hline(yintercept=-log(0.05/nrow(td_xf)), color="#BBBBBB") +
  ylab("-log(p Female)") +
  ggtitle("X Chromosome") +
  ylim(c(0,15)) +
  theme_classic(base_size=16) +

 #  theme_light() +
  theme(plot.title = element_text(hjust=0.5),
        plot.subtitle = element_text(hjust=0.5, face="italic"),
```

```
        legend.position = "none",
        strip.text = element_text(face="bold", size=12),
        panel.grid = element_blank(),
        axis.text.x = element_blank(),
        axis.title.x = element_blank())
```

```
## Joining, by = "probeID"
```

```
plot_manM <- td_xm %>%
  left_join(probeInfo %>% dplyr::select(probeID, pos)) %>%
  ggplot(aes(x=pos, y=-log(p.value))) +
  geom_point(alpha=0.2) +
  geom_hline(yintercept=-log(0.05/nrow(td_xm)),  color="#BBBBBB") +
  scale_y_reverse(lim=c(15,0)) +
  xlab("hg19 coordinate") +
  ylab("-log(p Male)") +
  theme_classic(base_size=16) +

 #  theme_light() +
  theme(plot.title = element_text(hjust=0.5),
        plot.subtitle = element_text(hjust=0.5, face="italic"),
        legend.position = "none",
        strip.text = element_text(face="bold", size=12),
        panel.grid = element_blank())
```

```
## Joining, by = "probeID"
```

```
plot_manhattan_x <- plot_grid(plot_manF, plot_manM, ncol=1, nrow=2)
```

```
## Warning: Removed 1 rows containing missing values (geom_point).
```

```
plot_manhattan_y <- td_ym %>%
  left_join(probeInfo %>% dplyr::select(probeID, pos)) %>%

  ggplot(aes(x=pos, y=-log(p.value))) +
  geom_point(alpha=0.2) +
  geom_hline(yintercept=-log(0.05/nrow(td_ym)), color="#BBBBBB") +
  ylab("-log(p Male)") +
  xlab("hg19 coordinate") +
  ggtitle("Y Chromosome") +
  ylim(c(0,15)) +
  theme_classic(base_size=16) +

 #  theme_light() +
  theme(plot.title = element_text(hjust=0.5),
        plot.subtitle = element_text(hjust=0.5, face="italic"),
        legend.position = "none",
        strip.text = element_text(face="bold", size=12),
        panel.grid = element_blank())
```

```
## Joining, by = "probeID"
```

```
bottom <- plot_grid(plot_manhattan_y, plot_xHit, ncol=2, nrow=1, axis="b", align="v", labels=c("B.", "C
```

5.3 Manuscript figure

```
fig_aCA_composite <- plot_grid(plot_manhattan_x, bottom, nrow=2, ncol=1, labels=c("A."), rel_heights=c(
fig_aCA_composite
```

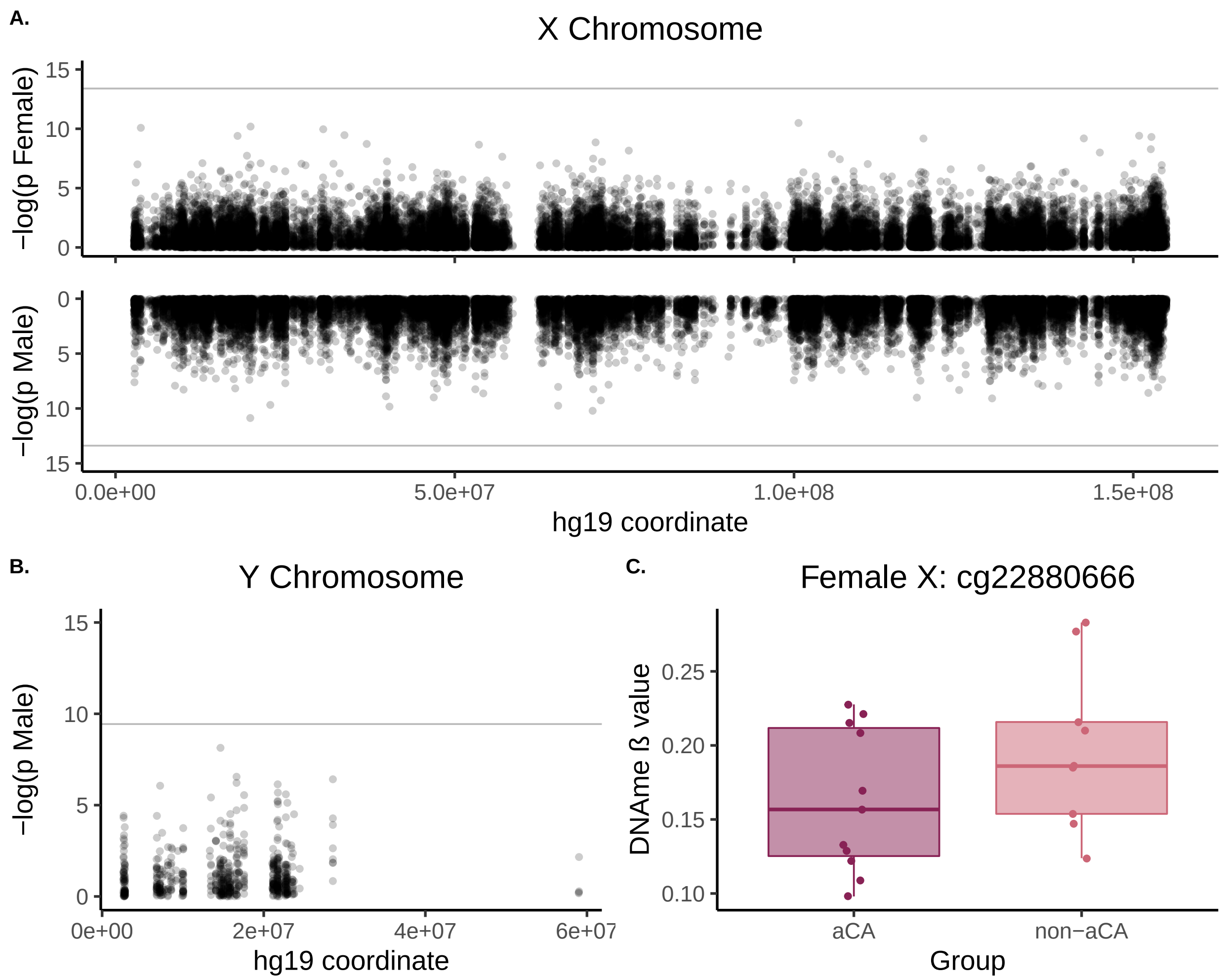

6.0 Session Info

```
sessionInfo()
```

```
## R version 4.1.2 (2021-11-01)
## Platform: x86_64-w64-mingw32/x64 (64-bit)
## Running under: Windows Server x64 (build 14393)
##
## Matrix products: default
```

```
##
## locale:
## [1] LC_COLLATE=English_Canada.1252  LC_CTYPE=English_Canada.1252
## [3] LC_MONETARY=English_Canada.1252 LC_NUMERIC=C
## [5] LC_TIME=English_Canada.1252
##
## attached base packages:
## [1] parallel  stats4    stats     graphics  grDevices utils     datasets
## [8] methods   base
##
## other attached packages:
##  [1] IlluminaHumanMethylationEPICmanifest_1.0.0
##  [2] IlluminaHumanMethylationEPICanno.ilm10b4.hg19_0.6.0
##  [3] cowplot_1.1.1
##  [4] wateRmelon_2.0.0
##  [5] illuminaio_0.36.0
##  [6] IlluminaHumanMethylation450kanno.ilmn12.hg19_0.6.0
##  [7] ROC_1.70.0
##  [8] methylumi_2.40.1
##  [9] FDb.InfiniumMethylation.hg19_2.2.0
## [10] org.Hs.eg.db_3.14.0
## [11] TxDb.Hsapiens.UCSC.hg19.knownGene_3.2.2
## [12] GenomicFeatures_1.46.1
## [13] AnnotationDbi_1.56.2
## [14] reshape2_1.4.4
## [15] scales_1.1.1
## [16] limma_3.50.0
## [17] sesameData_1.12.0
## [18] rmarkdown_2.11
## [19] ExperimentHub_2.2.0
## [20] AnnotationHub_3.2.0
## [21] BiocFileCache_2.2.0
## [22] dbplyr_2.1.1
## [23] minfi_1.40.0
## [24] bumphunter_1.36.0
## [25] locfit_1.5-9.4
## [26] iterators_1.0.13
## [27] foreach_1.5.1
## [28] Biostrings_2.62.0
## [29] XVector_0.34.0
## [30] SummarizedExperiment_1.24.0
## [31] MatrixGenerics_1.6.0
## [32] matrixStats_0.61.0
## [33] GenomicRanges_1.46.1
## [34] GenomeInfoDb_1.30.0
## [35] IRanges_2.28.0
## [36] S4Vectors_0.32.2
## [37] lumi_2.46.0
## [38] GEOquery_2.62.1
## [39] Biobase_2.54.0
## [40] BiocGenerics_0.40.0
## [41] ewastools_1.7
## [42] biobroom_1.26.0
## [43] broom_0.7.10
```

```
## [44] here_1.0.1
## [45] forcats_0.5.1
## [46] stringr_1.4.0
## [47] dplyr_1.0.7
## [48] purrr_0.3.4
## [49] readr_2.1.0
## [50] tidyr_1.1.4
## [51] tibble_3.1.6
## [52] ggplot2_3.3.5
## [53] tidyverse_1.3.1
##
## loaded via a namespace (and not attached):
##   [1] utf8_1.2.2                  tidyselect_1.1.1
##   [3] RSQLite_2.2.8               grid_4.1.2
##   [5] BiocParallel_1.28.1         munsell_0.5.0
##   [7] codetools_0.2-18            preprocessCore_1.56.0
##   [9] nleqslv_3.3.2               withr_2.4.2
##  [11] colorspace_2.0-2            filelock_1.0.2
##  [13] highr_0.9                   knitr_1.36
##  [15] rstudioapi_0.13             labeling_0.4.2
##  [17] tableone_0.13.0             GenomeInfoDbData_1.2.7
##  [19] farver_2.1.0                bit64_4.0.5
##  [21] rhdf5_2.38.0                rprojroot_2.0.2
##  [23] vctrs_0.3.8                 generics_0.1.1
##  [25] xfun_0.28                   R6_2.5.1
##  [27] bitops_1.0-7                rhdf5filters_1.6.0
##  [29] cachem_1.0.6                reshape_0.8.8
##  [31] DelayedArray_0.20.0         assertthat_0.2.1
##  [33] promises_1.2.0.1            BiocIO_1.4.0
##  [35] gtable_0.3.0                affy_1.72.0
##  [37] rlang_0.4.12                genefilter_1.76.0
##  [39] splines_4.1.2               rtracklayer_1.54.0
##  [41] BiocManager_1.30.16         yaml_2.2.1
##  [43] modelr_0.1.8                backports_1.4.0
##  [45] httpuv_1.6.3                tools_4.1.2
##  [47] nor1mix_1.3-0               affyio_1.64.0
##  [49] ellipsis_0.3.2              RColorBrewer_1.1-2
##  [51] proxy_0.4-26                siggenes_1.68.0
##  [53] Rcpp_1.0.7                  plyr_1.8.6
##  [55] sparseMatrixStats_1.6.0     progress_1.2.2
##  [57] zlibbioc_1.40.0             RCurl_1.98-1.5
##  [59] prettyunits_1.1.1           openssl_1.4.5
##  [61] zoo_1.8-9                   haven_2.4.3
##  [63] survey_4.1-1                fs_1.5.0
##  [65] magrittr_2.0.1              data.table_1.14.2
##  [67] reprex_2.0.1                hms_1.1.1
##  [69] mime_0.12                   evaluate_0.14
##  [71] xtable_1.8-4                XML_3.99-0.8
##  [73] mclust_5.4.8                readxl_1.3.1
##  [75] compiler_4.1.2              biomaRt_2.50.1
##  [77] KernSmooth_2.23-20          crayon_1.4.2
##  [79] htmltools_0.5.2             mgcv_1.8-38
##  [81] later_1.3.0                 tzdb_0.2.0
##  [83] lubridate_1.8.0             DBI_1.1.1
```

```
##  [85] MASS_7.3-54                 rappdirs_0.3.3
##  [87] Matrix_1.3-4                mitools_2.4
##  [89] cli_3.1.0                   quadprog_1.5-8
##  [91] igraph_1.2.8                pkgconfig_2.0.3
##  [93] GenomicAlignments_1.30.0    xml2_1.3.2
##  [95] annotate_1.72.0             rngtools_1.5.2
##  [97] multtest_2.50.0             beanplot_1.2
##  [99] rvest_1.0.2                 doRNG_1.8.2
## [101] scrime_1.3.5                digest_0.6.28
## [103] base64_2.0                  cellranger_1.1.0
## [105] DelayedMatrixStats_1.16.0   restfulr_0.0.13
## [107] curl_4.3.2                  shiny_1.7.1
## [109] Rsamtools_2.10.0            rjson_0.2.20
## [111] lifecycle_1.0.1             nlme_3.1-153
## [113] jsonlite_1.7.2              Rhdf5lib_1.16.0
## [115] askpass_1.1                 fansi_0.5.0
## [117] labelled_2.9.0              pillar_1.6.4
## [119] lattice_0.20-45             KEGGREST_1.34.0
## [121] fastmap_1.1.0               httr_1.4.2
## [123] survival_3.2-13             interactiveDisplayBase_1.32.0
## [125] glue_1.5.0                  png_0.1-7
## [127] BiocVersion_3.14.0          bit_4.0.4
## [129] class_7.3-19                stringi_1.7.5
## [131] HDF5Array_1.22.1            blob_1.2.2
## [133] memoise_2.0.0               e1071_1.7-9
```
